# Evaluation of energy density and levels of fat, sugar, and salt in processed and ultra-processed food with existing and novel nutrient profiling models to identify unhealthy food and drink: cross sectional analysis of a nationally representative database of food and drink items

**DOI:** 10.1136/bmjmed-2025-001750

**Published:** 2026-11-10

**Authors:** Samuel J Dicken, Dolly RZ van Tulleken, Adrian Brown, Fabio S Gomes, Christoffer van Tulleken

**Affiliations:** 1 Centre for Obesity Research, University College London, London, UK; 2 Department of Behavioural Science and Health, University College London, London, UK; 3 National Institute for Health and Care Research (NIHR) University College London Hospitals Biomedical Research Centre, London, UK; 4 Medical Research Council (MRC) Epidemiology Unit, University of Cambridge, Cambridge, UK; 5 Pan American Health Organization (PAHO)/World Health Organization (WHO), Washington, DC, USA; 6 Division of Infection and Immunity, University College London, London, UK

**Keywords:** Health policy, Nutritional sciences, Public health

## Abstract

**Objectives:**

To assess the proportions of processed and ultraprocessed products in the UK that are high in fat, salt, and sugar, and energy density, defined by two systems used to regulate unhealthy food products in the UK (Food Standards Agency (FSA) nutrient profiling model (NPM) and front-of-pack traffic lights labelling system), and compared with UK dietary guidance based on new NPMs.

**Design:**

Cross sectional analysis of a nationally representative database of food and drink items.

**Setting:**

UK National Diet and Nutrition Survey database.

**Population:**

Food and drink items (262 processed products and 1636 ultraprocessed products) from the National Diet and Nutrition Survey database.

**Main outcome measures:**

Proportion of processed and ultraprocessed products identified as unhealthy based on three Eatwell Guide NPMs, the FSA NPM, and the front-of-pack traffic lights labelling system. Three new nutrient profile models based on UK dietary guidance were constructed to identify excess saturated fat, free sugar, sodium, total fat, and energy density in processed and ultraprocessed products: NPM1 identified excess saturated fat, free sugar, and sodium; NPM2 identified excess saturated fat, free sugar, sodium, and total fat; and NPM3 identified excess saturated fat, free sugar, sodium, total fat, and energy density.

**Results:**

The proportion of ultraprocessed products identified as unhealthy differed significantly (P<0.05) between the NPMs. The FSA NPM identified as unhealthy 57.6% of ultraprocessed products (95% confidence interval 55.2% to 60.0%), whereas the traffic lights labelling system identified fewer than half of all ultraprocessed products as unhealthy (48.2%, 45.8% to 50.6%). NPM1 identified 91.5% (95% CI 90.2% to 92.9%) of all ultraprocessed products as unhealthy, model No 2 identified 94.9% (93.9% to 96.0%), and model No 3 identified 98.7% (98.2% to 99.3%) of all ultraprocessed products.

**Conclusions:**

Food and drink products that, according to UK government dietary guidance, contain excess amounts of harmful nutrients are not identified by current systems for regulating UK food and drink (FSA NPM and front-of-pack traffic lights labelling system). The most conservative new NPM, based on current UK government dietary guidance, identified significantly greater proportions of processed and ultraprocessed products as unhealthy than the current two systems. These findings have important implications for food policy and the appropriateness of UK dietary guidance to identify unhealthy products.

**WHAT IS ALREADY KNOWN ON THIS TOPIC**

**WHAT THIS STUDY ADDS**

- About half of processed and ultraprocessed products were identified as unhealthy by the current FSA NPM and the front-of-pack traffic lights labelling system
- More than 90% of processed and ultraprocessed products were identified as unhealthy according to current UK government dietary guidance, significantly more than the proportions identified with the FSA NPM (also known as high fat, salt, and sugar), and front-of-pack multiple traffic lights labelling system
- Products with excess content of harmful nutrients were not all identified by the current food and drink regulations (FSA NPM and traffic lights labelling system)

**HOW THIS STUDY MIGHT AFFECT RESEARCH, PRACTICE, OR POLICY**

- This study highlights regulatory loopholes and misalignment of the FSA NPM and the traffic lights labelling system with UK dietary guidance in identifying unhealthy food and drink products
- This highlights the possible benefit for all stakeholders in having a single system using agreed thresholds aligned with UK dietary guidance to identify unhealthy products for policy and regulation

## Introduction

In 2017, poor diet overtook tobacco as the leading global cause of premature mortality,^1,2^ with the prevalence of diet related non-communicable disease in the UK having rapidly risen in recent decades.^1^ Diets linked to poor health contain high proportions of nutritionally poor, energy dense foods,^1,3^ high in saturated fat, free sugar, and sodium, and low in fruit, vegetables, and fibre.^1,3^ In contrast, diets linked with improved health outcomes contain high proportions of whole and minimally processed foods, and are high in fibre and micronutrients and low in saturated fat, free sugar, and sodium.^1,4,5^ Traditional definitions of unhealthy food therefore typically focus on nutrient content.

The UK government uses three separate systems to define unhealthy food products and diet for regulation and public dietary advice: official UK government dietary guidance in the Eatwell Guide,^6^ the Food Standards Agency (FSA) nutrient profile model,^7^ and the front-of-pack traffic lights on the labels of prepackaged products.^8^ Table 1 provides a detailed overview of these three systems, including their application and purpose.

**Table 1:** Overview of the Eatwell Guide, Food Standards Agency nutrient profiling model, and front-of-pack multiple traffic lights labelling system NPM

System	Description	Calculation or guidance and supporting evidence	Application to food and drink	Purpose and use in public health
Eatwell Guide	UK food-based dietary guidelines on how much should be consumed from different food groups for a healthy balanced diet (eg, a woman aged 19-65 years should consume 2100 kcal/day).^6^ Originally developed in 1994 as the Balance of Good Health, then updated to the Eatwell plate in 2007, and revised to the current Eatwell Guide in 2016^70^	Recommended upper limits on energy, fat, saturated fat, free sugar, and sodium intake, and minimum recommendations for fruit and vegetables, and fibre intake. Basing meals around starchy carbohydrates and opting for wholegrain or high fibre options, eating more beans and pulses, two weekly portions of fish (one of which is oily), and limiting intake of processed and red meat. Based on well evidenced^70^ independent recommendations by the Scientific Advisory Committee on Nutrition,^9,10^ aligned with international dietary guidance, including the World Health Organization^3^ and broadly similar across European Union nations^54^	Broad. All food and drink items	Used for public consumer understanding; not used in mandatory legislation to regulate unhealthy food. “A policy tool used to define government recommendations on eating healthily and achieving a balanced diet”^6^
FSA NPM	Developed in 2004-05 to help the media and communications regulator, Ofcom, identify “less healthy”^7^ food and drink items (known as high fat, salt, and sugar), and used in food marketing regulations since 2007	An overall score for food and drink based on summing the quantities of favourable (fruit, vegetables, and nuts, fibre, and protein) and unfavourable (energy, saturated fat, total sugar, and sodium) nutrients.^7^ Products that exceed a particular score are designated as less healthy and high in fat, salt, or sugar for marketing legislation. The calculation is based on the composition of existing products,^58,59^ and not based on the latest UK government dietary guidance (in the Eatwell Guide) for free sugar and fibre. A revised version was developed in 2018 (FSA NPM 2018)^36^ and published in 2026,^37^ to reflect current UK dietary guidance, but is currently not used	Narrow. Only applies to 13 specific categories of packaged goods (online supplemental table 1 has the list of categories)^7^	Not used in voluntary policy; used only in mandatory legislation to regulate unhealthy food marketing restrictions on television, online advertisements, and promotions by price and volume.^71^ New UK regulations on online and TV advertisements came into force on 5 January 2026^72^
Front-of-pack multiple traffic lights labelling system	Developed in 2006 by the Department of Health and the FSA to advise consumers on the nutrient profile of all prepackaged food and drink items at the point of purchase,^8^ with the current scheme introduced in 2013	Green, amber, or red colours are assigned to the front of pack to indicate low, medium, or high levels of fat, saturated fat, salt, or total sugar content, respectively.^8^ The low cut-off value (green) is based on the “low” nutrition claim for fat, saturates, total sugars, and salt in the European Union Nutrition and Health Claims Regulation legislation 1924/2006.^73^ The rationale behind the high cut-off value (red), which is based on a percentage of reference intakes per 100 g or per portion, is unclear^8^	Broad. All prepackaged food and drink items	Not used in mandatory policy; used only voluntarily by manufacturers on the labels of prepackaged products for consumer focused labelling at point of purchase in the retail environment (back-of-pack nutrition labelling is mandatory)

1 kcal=4.18 kJ.

FSA, Food Standards Agency; NPM, nutrient profiling model.

UK government dietary recommendations in the Eatwell Guide are intended to inform food policy and regulation, and are based on well evidenced independent recommendations by the Scientific Advisory Committee on Nutrition,^9,10^ and are aligned with international guidance (eg, World Health Organization (WHO)).^3^ Despite similarities in how these systems characterise unhealthy food and drink items, inconsistencies exist. First, the nutrient thresholds of the FSA NPM and front-of-pack traffic lights labelling system to define unhealthy food items are misaligned with UK government dietary recommendations; second, entire categories of food which typically have high levels of saturated fat, salt, or sugar, or combinations of these, are exempt from the FSA NPM, including savoury pastries and chocolate spreads^11^; and third, the FSA NPM is inconsistent with the traffic lights labelling system (products labelled with red traffic lights are not all classified as high fat, salt, and sugar by the FSA NPM). Online supplemental table 2 outlines these inconsistencies in more detail. As a result, products rated as unhealthy by one system can be rated as not unhealthy by another, and vice versa. Hence many products considered unhealthy according to UK government dietary guidance might not be identified by the other two systems. Thus these products may remain unregulated and heavily marketed, and could be perceived as healthy by consumers, but so far no study has directly tested these inconsistencies.

Although definitions of unhealthy food have typically been nutrient based, recent shifts towards the rise in diet related disease in developed countries have been attributed to changes in the food system beyond nutrient composition alone.^12^ These links between changes in the food system and diet related disease are consistent with evidence linking ultraprocessed foods or products, as defined by the Nova classification, to poor health outcomes.^13^ The Nova classification considers the purpose and extent of processing, and defines ultraprocessed products as being industrially manufactured prepackaged foods,^14^ including ingredients not used at home, such as cosmetic additives, modified starches, or isolated proteins.^15,16^ Ultraprocessed products are designed with the primary purpose of maximising sales and profit. The Nova classification is used by WHO and the Food and Agriculture Organisation of the United Nations,^17,18^ and guidance to reduce ultraprocessed products is recommended by the American Heart Association^19^ and several countries' national dietary guidelines.^20^

UK dietary guidance, however, does not formally advise on the purpose and extent of industrial food processing,^21^ and is not considered in the FSA NPM or the front-of-pack traffic lights labelling system. The Nova classification is controversial and has been criticised for being too ambiguous, difficult to implement, and offering limited value in addition to existing regulatory tools.^22^ Members of the UK government have stated that the definition of ultraprocessed products is too broad for policy use, and that ultraprocessed products are already well described by existing legislation, including high fat, salt, and sugar according to the FSA NPM, or UK dietary guidance.^23–25^ But so far no study has directly tested this claim. Furthermore, no study has assessed the relative ability of UK government dietary recommendations, the FSA NPM, or the traffic lights labelling system to identify less healthy processed or ultraprocessed products, as defined by the Nova classification. Consistency between these systems has also not been evaluated.

Therefore, the aims of this study were to: calculate the proportion of processed and ultraprocessed products in the UK identified as unhealthy, according to the current FSA NPM and front-of-pack traffic lights labelling system; develop new NPMs based on UK dietary recommendations in the Eatwell Guide; and compare the proportions of processed and ultraprocessed products identified as unhealthy according to the current FSA NPM and front-of-pack traffic lights labelling system with the new NPMs based on the Eatwell Guide.

## Methods

This study was a cross sectional analysis of a nationally representative database of food and drink items consumed in the UK. The methods section defines the food and drink items in scope for the analysis, and describes the rationale and calculation of existing NPMs as well as new NPMs based on UK dietary guidance. Reporting conducted according to STROBE guidance (online supplemental file 2).

### Food and drink items in scope

No publicly available database exists of all food and drink products in the UK, nor a database of products in scope for regulation by the FSA NPM.^11^ Therefore, a nationally representative database of all food and drink products in the UK was used to identify processed and ultraprocessed products according to the Nova classification,^16^ as a proxy for prepacked items in the UK (and similar to the Pan American Health Organization^26^) and for identifying the proportion of processed and ultraprocessed products as unhealthy by NPMs.

Nutrient composition data of processed and ultraprocessed products from year 12 of the National Diet and Nutrition Survey were used. The National Diet and Nutrition Survey is a repeated cross sectional survey that has collected dietary intake from a nationally representative sample of the UK population, aged ≥1.5 years and living in private households, since 2008.^27^ Until 2019 (years 1-11), dietary assessment was conducted with four day food diaries across consecutive days. In 2019-20 (year 12), diaries were replaced with four non-consecutive, multiple pass, 24 hour dietary recalls with Intake24 (https://intake24.co.uk), with an updated food and drink database to reflect the change in diet assessment.^28^ The rationale and subsequent updates to the food and drink database are reported elsewhere.^28^

The list of food and drink items in the National Diet and Nutrition Survey year 12 database were obtained from the Intake24 team, and the matching nutrient databank was obtained from the UK Data Service (https://beta.ukdataservice.ac.uk). The values in the nutrient databank are the latest publicly available data on the nutrient content for each food and drink item. Values are obtained from multiple sources, including the UK Composition of Foods Integrated Dataset,^29^ the FSA Food Recipes Database,^30^ and manufacturer data from food labels and the internet. Nutrient values are assigned to all foods, with no missing values. All values are inspected before their addition to the databank,^31^ and for nutrients lacking reliable data, values from similar foods are extrapolated.

All processed and ultraprocessed products were used as products in scope, because directly mapping in-scope categories of the FSA NPM to the food and drink groups of the National Diet and Nutrition Survey database, or directly identifying specific exempt items from within in-scope categories, was not possible. This approach to prioritise sensitivity over specificity for capturing items for consideration is similar to previous approaches applying the FSA NPM to nutrient databases.^32^

### Identifying processed and ultraprocessed products by Nova classification

Food and drink items from the year 12 food and drink database were coded according to the Nova classification (online supplemental table 3 lists the definitions),^16^ as described elsewhere.^33^ Nova classification coding was conducted for individual items with the authors masked to the nutrient databank. Initial coding was conducted by one of the authors (SJD) and then discussed with a registered senior specialist dietitian (AB). Coding was determined with multiple sources, including the item name, subgroup, representative products from UK supermarkets, and the assigned Nova group of the corresponding item in the National Diet and Nutrition Survey food and drink database from years 1-11.^34^ Both authors agreed on the Nova classification for ambiguous food items and food groups.

Of the 3105 items in the database, 109 items were designated as outside of the Nova classification (eg, fish oil supplements and multivitamins) and 16 items had no corresponding item in the National Diet and Nutrition Survey nutrient databank, as previously reported.^33^ Alcoholic drinks were excluded from this analysis because of other specific regulations (n=35).^31^ Of the 2945 remaining items, 1636 were classified as ultraprocessed products, 262 as processed products, 61 as processed culinary ingredients, and 986 were minimally processed foods.

### Identifying unhealthy processed and ultraprocessed products

The proportion of processed and ultraprocessed products identified as unhealthy were calculated according to the FSA NPM algorithms, the front-of-pack traffic lights labelling system, and three new NPMs based on the Eatwell Guide.

#### Food Standards Agency nutrient profiling models

The FSA NPM is a binary system that classifies food and drink items as “healthy” or “less healthy”.^7,35^ The scoring system balances positive points for energy, saturated fat, total sugar, and sodium, and negative points for fruit, vegetables, and nut content, fibre, and protein, to give a final score.^7,35^ A maximum of 10 “A” points is awarded each for energy, saturated fat, total sugar, and salt, with greater points with increasing content. A maximum of five “C” points is awarded each for protein, fibre, and fruit, vegetables, and nut content, with greater points with increasing content. The final score is then calculated by subtracting the total number of “C” points from the total number of “A” points (unless a food is scored >10 “A” points and <5 points for fruit, vegetables, and nuts, where the protein score is not included).^7,35^ A food is identified as “less healthy” with a score of ≥4, and a drink as “less healthy” with a score of ≥1. Although the FSA NPM can be applied to any product, in UK legislation the model is applied to 13 specific product categories which exclude minimally processed foods and processed culinary ingredients (online supplemental table 1). “Less healthy” products identified within one of the specific product categories are regulated by marketing restrictions under the “Advertising (Less Healthy Food Definitions and Exemptions) Regulations 2024”.^11^

A revision of the FSA NPM in 2018 modified the score to reflect updated UK dietary guidance (model 2018),^36^ which was published in 2026^37^ but is currently not used in policy. The FSA NPM 2018 includes a change to the use of free sugars instead of total sugars, aiming for <5% of energy from free sugars,^36^ and increasing fibre “C” points up to a maximum of eight, based on current UK dietary guidance to consume at least 30 g/day of fibre.^36^ Processed and ultraprocessed products were identified as unhealthy according to current FSA NPM guidance (model 2004-05) and the revised 2018 model, based on the scoring definition of “less healthy”.

#### Front-of-pack traffic lights labelling system

The traffic lights scheme defines thresholds where each nutrient (fat, saturated fat, total sugar, and sodium) can be assigned a red, amber, or green colour to indicate a high, medium, or low amount, respectively (per 100 g or per portion). Items with low content for a specific nutrient are coded green, moderate content as amber, and high content as red (online supplemental table 4 shows the threshold values). For comparability, items were coded per 100 g for both processed and ultraprocessed food and drink items (which was assumed to be equivalent to 100 mL of drink). Drink items included lower cut-off values for red colour coding per 100 g of drink, according to the guidance from the traffic lights system. Based on evidence indicating that UK consumers more readily avoid red traffic lights over choosing green traffic lights when identifying healthier products,^38,39^ processed and ultraprocessed products were identified as unhealthy, according to guidance from the traffic lights labelling system, on the presence of one or more red traffic lights across any of the four nutrients, as previously used.^33,40^

#### New Eatwell Guide nutrient profiling models

Three new NPMs based on current UK dietary guidance were constructed.^6,41–43^ The NPMs were developed to directly translate UK government dietary guidance to food and drink items by using the Pan American Health Organization and WHO approach of proportionally scaling diet level intake goals to apply thresholds to products.^26^ UK dietary guidance includes recommendations for no more than 35%, 10%, 2%,^43^ and 5% of total energy intake from total fat, saturated fat, trans fat, and free sugars, respectively, and no more than 6 g/day of salt (table 2). These thresholds were used to construct the NPMs.

**Table 2:** UK dietary guidance recommendations^6,41–43,46,5^

Eatwell Guide component	Guidance
Energy	≤125 kcal/100 g (Food Standards Scotland guidance)^46^
Total fat	≤35% of energy intake
Saturated fat	≤10% of total energy intake
Sodium	≤2363 mg/day sodium (6 g/day salt)^42^
Free sugars	≤5% of total energy intake
Trans fat	≤2% of total food energy intake^43^

See also Scheelbeek et al.

1 kcal=4.18 kJ.

The first NPM was based on thresholds for saturated fat, free sugar, and sodium (NPM1) to align with unfavourable nutrients (excluding energy) used in the FSA NPM while accounting for current UK dietary guidance focusing on free sugars and not total sugars. The second NPM was based on thresholds for saturated fat, free sugars, sodium, and total fat (NPM2) to align with nutrients used in the traffic lights labelling system. The third NPM was based on thresholds for saturated fat, free sugars, sodium, total fat, and energy density (NPM3). Energy was included given the evidence linking energy density with energy over-consumption, obesity, and obesity related non-communicable disease,^44,45^ and the energy intake recommendations in UK dietary guidance.^6^ The energy threshold in NPM3 was from the Food Standards Scotland Revised Dietary Goals for Scotland,^46^ which is based on the Eatwell Guide and dietary recommendations of the Scientific Advisory Committee on Nutrition. This threshold provides a non-consumer facing dietary energy density goal of ≤125 kcal (523 kJ)/100 g.^46^

Processed and ultraprocessed products were identified as unhealthy if the threshold for any nutrient within each of the three Eatwell Guide NPMs was exceeded, and additionally for energy in NPM3. The threshold for trans fat was also added to NPM2 and NPM3 in a sensitivity analysis. Table 3 summarises the six NPMs used in this analysis.

**Table 3:** Summary of nutrient profiling models for identifying unhealthy products

	Traffic lights labelling system*	FSA NPM 2004-05†	FSA NPM 2018‡	NPM1§	NPM2¶	NPM3**
Scope of food and drink items, subject to the application of each nutrient profiling model in the analysis	All processed or ultraprocessed products	All processed or ultraprocessed products	All processed or ultraprocessed products	All processed or ultraprocessed products	All processed or ultraprocessed products	All processed or ultraprocessed products
Identification of product as unhealthy	Exceeding one or more of the nutrient thresholds below	Food: overall score ≥4 points†† Drink: overall score ≥1 points††	Food: overall score ≥4 points†† Drink: overall score ≥1 points††	Exceeding one or more of the nutrient thresholds below	Exceeding one or more of the nutrient thresholds below	Exceeding one or more of the nutrient or energy thresholds below
Fat	Food: >17.5 g/100 g Drink: >8.75 g/100 mL				>35 kcal/100 kcal	>35 kcal/100 kcal
Saturated fat	Food: >5 g/100 g Drink: >2.5 g/100 mL	As part of algorithm††	As part of algorithm††	>10 kcal/100 kcal	>10 kcal/100 kcal	>10 kcal/100 kcal
Sugar	Food: >22.5 g of total sugar/100 g Drink: >11.25 g of total sugar/100 mL	Total sugar; as part of algorithm††	Total sugar; as part of algorithm††	>5 kcal from free sugars/100 kcal	>5 kcal from free sugars/100 kcal	>5 kcal from free sugars/100 kcal
Sodium	Food: >600 mg/100 g Drink: >300 mg/100 mL	As part of algorithm††	As part of algorithm††	>120 mg/100 kcal >360 mg/100 g or 100 mL	>120 mg/100 kcal >360 mg/100 g or 100 mL	>120 mg/100 kcal >360 mg/100 g or 100 mL
Energy		As part of algorithm††	As part of algorithm††			>125 kcal/100 g or 100 mL
Fruit, vegetables, and nuts		As part of algorithm††	As part of algorithm††			

1 kcal=4.18 kJ.

* Department of Health guidance for application on front-of-package labelling.

† UK Food Standards Agency nutrient profiling model for regulation of children's advertising, 2004-05.

‡ Revised UK Food Standards Agency nutrient profiling model for regulation of children's advertising from 2018.

§ Criteria based on UK Public Health recommended dietary intake for saturated fats, sugar, and sodium. –

¶ Criteria based on UK Public Health recommended dietary intake for total fat, saturated fat, sugar, and sodium. –

** Criteria based on UK Public Health recommended dietary intake for energy density, total fat, saturated fat, sugar, and sodium. –

†† Food Standards Agency nutrient profiling model summary score algorithm considers energy, saturated fat, total sugar, sodium, fibre, and protein content in 100 g of food or drink items, as well as percentage of fruit, vegetable, and nut content.

FSA, Food Standards Agency; NPM, nutrient profiling model.

### Statistical analysis

Categorical variables are described as counts and percentages. The proportion of processed and ultraprocessed products identified as unhealthy according to all NPMs are reported with 95% confidence intervals (CIs). The proportion of processed and ultraprocessed products identified as unhealthy are then reported according to each nutrient or energy threshold in the traffic lights and Eatwell Guide NPMs (the FSA NPM is an overall score to identify unhealthy products and is therefore not reported).

The performance of each NPM in identifying processed and ultraprocessed products as unhealthy and not unhealthy was compared against all other NPMs with Wald statistics for homogeneity, applying Rao-Scott first order and second order corrections. To quantify the performance of theNPMs in identifying unhealthy ultraprocessed products, we used prevalence ratios with 95% CIs to compare the difference in magnitude in the proportion of ultraprocessed products identified as unhealthy by each NPM.

To assess the overlap between each NPM and the Nova classification of ultraprocessed products, Cohen's κ with 95% CIs was used to compare the agreement between the proportion of ultraprocessed products identified as unhealthy by each NPM and the total number of ultraprocessed products. Non-homogeneous log linear models were then used to rank each NPM according to their level of agreement with the Nova classification of ultraprocessed products.^47^ All tests were two sided and significance was set at P<0.05. Analyses were conducted in R, version 4.4.1.

### Patient and public involvement

This study analysed a nutrient database and did not involve participants. Therefore, patients and the public were not involved in the design, planning, nor conduct of this research. Results will be shared with the public through media and press release.

## Results

Table 4 and figure 1 report the proportion of processed and ultraprocessed products identified as unhealthy by each NPM. The proportion of ultraprocessed products identified as unhealthy differed significantly between all NPMs (P<0.05). NPM3 identified the greatest proportion of ultraprocessed products as unhealthy (98.7%, 95% CI 98.2% to 99.3%), followed by NPM2 (94.9%, 93.9% to 96.0%), NPM1 (91.5%, 90.2% to 92.9%), FSA NPM 2018 (68.3%, 66.1% to 70.6%), FSA NPM 2004-05 (57.6%, 55.2% to 60.0%), and front-of-pack traffic lights labelling system (48.2%, 45.8% to 50.6%) (figure 1). The proportion of processed products identified as unhealthy did not differ significantly between the three Eatwell Guide NPMs (NPM1 91.6%, 95% CI 88.2% to 95.0%; NPM2 93.1%, 90.1% to 96.2%; and NPM3 95.0%, 92.4% to 97.7), but was significantly greater than the proportion of processed products identified as unhealthy by the traffic lights labelling system (46.2%, 40.1% to 52.2%), FSA NPM 2004-05 (50.0%, 43.9% to 56.0%), and FSA NPM 2018 (55.0%, 48.9% to 61.0%) (P<0.05). The proportion of processed products identified as unhealthy by the traffic lights labelling system and both FSA NPMs did not differ significantly (table 4 and figure 1). The three Eatwell Guide NPMs identified significantly greater proportions of processed and ultraprocessed products as unhealthy than both FSA NPMs and the traffic lights labelling system (P<0.05).

**Table 4:** Percentage of processed and ultraprocessed products identified as unhealthy* in the UK according to each nutrient profiling model, with 95% confidence intervals

	Traffic lights labelling system	FSA NPM 2004-05	FSA NPM 2018	NPM1	NPM2	NPM3
Ultraprocessed products (n=1636)	48.2 (45.8 to 50.6)†	57.6 (55.2 to 60.0)‡	68.3 (66.1 to 70.6)§	91.5 (90.2 to 92.9)¶	94.9 (93.9 to 96.0)**	98.7 (98.2 to 99.3)††
Processed products (n=262)	46.2 (40.1 to 52.2)†	50.0 (43.9 to 56.0)†	55.0 (48.9 to 61.0)†	91.6 (88.2 to 95.0)‡	93.1 (90.1 to 96.2)‡	95.0 (92.4 to 97.7)‡

Scope of food and drink products subject to the application of all NPMs are all processed or ultraprocessed products, or both.

Different symbols (*, †, ‡, §, ¶) within a row indicate significant differences in the proportion of ultraprocessed and processed products identified as unhealthy by each NPM (P<0.05).

* Food and drink items identified as having high amounts of at least one of nutrients or energy (NPMs 1, 2, and 3, and traffic lights labelling system), or categorised as less healthy (Food Standards Agency NPM 2004-05 and model 2018).

† P<0.05.

‡ P<0.05.

§ P<0.05.

¶ P<0.05.

** P<0.05.

†† P<0.05.

FSA, Food Standards Agency ; N/A, not available; NPM, nutrient profiling model.

**Figure 1:**
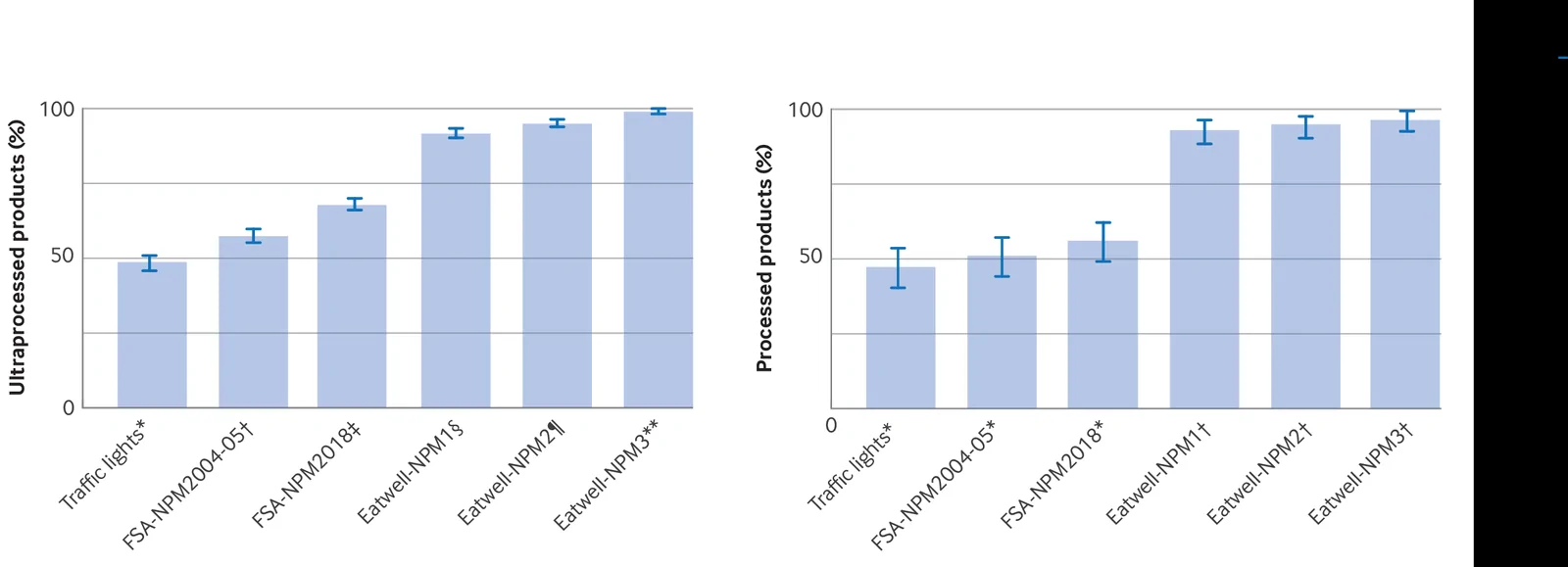
Percentage of ultraprocessed (left) and processed (right) products identified as unhealthy in the UK by nutrient profiling model (NPM), with 95% confidence intervals. Unhealthy products are food and drink products identified as containing high amounts of at least one of nutrients or energy (Eatwell Guide NPM1 (Eatwell-NPM1), Eatwell Guide NPM2 (Eatwell NPM2), Eatwell Guide NPM3 (Eatwell NPM3), and front-of-pack traffic lights labelling system (Traffic lights)), or categorised as less healthy (Food Standards Agency NPM 2004-05 (FSA NPM 2004-05) and Food Standards Agency NPM 2018 (FSA NPM 2018)). Different symbols (*, †, ‡, §, ¶) indicate significant differences in the proportion of ultraprocessed and processed products identified as unhealthy by each NPM (P<0.05)

Table 5 shows the proportion of processed and ultraprocessed products identified as unhealthy according to the specific parameter (nutrient or energy) and number of parameters in excess in the traffic lights labelling system and Eatwell Guide NPMs. Within each like-for-like parameter, the three Eatwell Guide NPMs identified a significantly greater proportion of processed and ultraprocessed products as unhealthy than the traffic lights labelling system (P<0.05), and significantly greater proportions of processed and ultraprocessed products as unhealthy based on containing two, three, or four parameters in excess (P<0.05). Compared with excess sugars, sodium, saturated fat, and fat, excess energy as an individual parameter identified the greatest proportion of processed products (69.1%, 95% CI 63.5% to 74.7%) and ultraprocessed products (74.3%, 72.1 to 76.4) as unhealthy. The addition of trans fat thresholds to NPM2 or NPM3 did not significantly change the proportion of processed or ultraprocessed products identified as unhealthy (data not shown).

**Table 5:** Percentage of processed and ultraprocessed products identified as unhealthy* in the UK according to the specific parameter in excess and the number of parameters in excess based on the front-of-pack traffic lights labelling system and Eatwell Guide nutrient profiling models (NPMs), with 95% confidence intervals

	Traffic lights labelling system	NPM1	NPM2	NPM3
Ultraprocessed products (n=1636)				
Excess sugars	22.7 (20.7 to 24.7)†	51.9 (49.5 to 54.3)‡	51.9 (49.5 to 54.3)‡	51.9 (49.5 to 54.3)‡
Excess sodium	12.1 (10.5 to 13.7)†	51.6 (49.2 to 54.0)‡	51.6 (49.2 to 54.0)‡	51.6 (49.2 to 54.0)‡
Excess saturated fat	28.6 (26.4 to 30.7)†	47.1 (44.7 to 49.6)‡	47.1 (44.7 to 49.6)‡	47.1 (44.7 to 49.6)‡
Excess fat	22.4 (20.4 to 24.5)†	N/A	48.7 (46.2 to 51.1)‡	48.7 (46.2 to 51.1)‡
Excess energy	N/A	N/A	N/A	74.3 (72.1 to 76.4)
≥1 parameter in excess (any nutrient or energy)	48.2 (45.8 to 50.7)†	91.5 (90.2 to 92.9)¶	94.9 (93.9 to 96.0)**	98.7 (98.2 to 99.3)††
≥2 parameters in excess (any nutrient or energy, or both)	25.9 (23.7 to 28.0)†	54.2 (51.8 to 56.7)‡	66.3 (64.0 to 68.6)§	86.6 (85.0 to 88.3)¶
≥3 parameters in excess (any nutrient or energy, or both)	11.6 (10.0 to 13.1)†	5.1 (4.0 to 6.1)‡	35.5 (33.2 to 37.8)§	55.8 (53.4 to 58.2)¶
≥4 parameters in excess (any nutrient or energy, or both)	0.1 (0.0 to 0.3)†	N/A	2.9 (2.1 to 3.7)‡	31.1 (28.8 to 33.3)§
5 parameters in excess	N/A	N/A	N/A	2.0 (1.3 to 2.6)
Processed products (n=262)				
Excess sugars	11.1 (7.3 to 14.9)†	29.4 (23.9 to 34.9)‡	29.4 (23.9 to 34.9)‡	29.4 (23.9 to 34.9)‡
Excess sodium	17.6 (13.0 to 22.2)†	50.4 (44.3 to 56.4)‡	50.4 (44.3 to 56.4)‡	50.4 (44.3 to 56.4)‡
Excess saturated fat	32.4 (26.8 to 38.1)†	61.8 (56.0 to 67.7)‡	61.8 (56.0 to 67.7)‡	61.8 (56.0 to 67.7)‡
Excess fat	24.8 (19.6 to 30.0)†	N/A	65.7 (59.9 to 71.4)‡	65.7 (59.9 to 71.4)‡
Excess energy	N/A	N/A	N/A	69.1 (63.5 to 74.7)
≥1 parameter in excess (any nutrient or energy)	46.2 (40.1 to 52.2)†	91.6 (88.2 to 95.0)‡	93.1 (90.1 to 96.2)‡	95.0 (92.4 to 97.7)‡
≥2 parameters in excess (any nutrient or energy, or both)	28.2 (22.8 to 33.7)†	48.1 (42.0 to 54.1)‡	71.0 (65.5 to 76.5)§	77.9 (72.8 to 82.9)§
≥3 parameters in excess (any nutrient or energy, or both)	11.5 (7.6 to 15.3)†	1.9 (0.3 to 3.6)‡	41.6 (35.6 to 47.6)§	63.4 (57.5 to 69.2)¶
≥4 parameters in excess (any nutrient or energy, or both)	0.0†	N/A	1.5 (0.0 to 3.0)‡	38.6 (32.7 to 44.4)§
5 parameters in excess	N/A	N/A	N/A	1.5 (0.0 to 3.0)

Different symbols (†, ‡, §, ¶, **, ††) within a row indicate significant differences in the proportion of ultraprocessed and processed products identified as unhealthy by each NPM (P<0.05).

* Food and drink identified as containing high amounts of at least one of nutrients or energy.

† P<0.05.

‡ P<0.05.

§ P<0.05.

¶ P<0.05.

** P<0.05.

†† P<0.05.

N/A, not available; NPM, nutrient profiling model.

Online supplemental table 5 reports the difference in magnitude in the proportion of ultraprocessed products identified as unhealthy between each NPM, and their ranking based on agreement with the Nova classification of ultraprocessed products. NPM3 identified as unhealthy more than twice as many ultraprocessed products than the traffic lights labelling system (prevalence ratio 2.05, 95% CI 1.95 to 2.15), and identified as unhealthy 71%, 45%, 8%, and 4% more ultraprocessed products than the FSA NPM 2004-05 (prevalence ratio 1.71, 1.64 to 1.79), FSA NPM 2018 (prevalence ratio 1.45, 1.40 to 1.49), NPM1 (prevalence ratio 1.08, 1.06 to 1.09), and NPM2 (prevalence ratio 1.04, 1.03 to 1.05), respectively.

## Discussion

### Principal findings

In this study, we compared the performance and consistency of existing UK food systems for classification of unhealthy food with three new NPMs based on UK dietary guidance (ie, NPM1 identifying excess saturated fat, free sugar, and sodium; NPM2 identifying excess saturated fat, free sugar, sodium, and total fat; and NPM3 identifying excess saturated fat, free sugar, sodium, total fat, and energy density). NPM1, the most conservative NPM, identified >91% of processed and ultraprocessed products as unhealthy and misaligned with UK dietary guidance. This finding is consistent with previous studies of NPMs based on WHO dietary guidance.^48,49^ By comparison, the FSA NPM 2004-05 identified 57% and 50%, the FSA 2018 model identified 68% and 55%, and the front-of-pack traffic lights labelling system identified 48% and 46% of processed and ultraprocessed products as unhealthy, respectively. These findings challenge the assertion that ultraprocessed products are currently identified by existing food legislation,^23–25^ and that the FSA NPM and the traffic lights labelling system are not adequately aligned with UK dietary guidance.

The traffic lights labelling system does not seem to have improved the healthiness of retail food purchases in the UK.^50^ There is limited evidence that traffic lights labelling system improves the healthiness of food purchases, even in comparison with no front-of-pack labelling, and data are inconsistent on whether the system is more effective than “high in” front-of-pack warning labels.^51–53^ This finding seems to be in agreement with our results that the traffic lights labelling system identified only half of processed and ultraprocessed products as unhealthy compared with the NPMs based on UK dietary guidance. Furthermore, despite the links between food and drink energy density and obesity related poor health,^44,45^ the traffic lights labelling system provides no colour coded guidance on energy content. The system also uses total sugars to profile food, not free sugars, as in UK dietary guidance,^6^ and labelling is voluntarily applied to minimally processed foods, such as nuts or whole fruit (eg, avocados), despite these foods being encouraged as part of a healthy diet in UK and international dietary guidance.^6,54^ NPM1, the most conservative NPM, identified 43-46% more (nearly twice as many) processed and ultraprocessed products as unhealthy than the traffic lights labelling system. This finding is in line with previous data showing poor overlap between ultraprocessed products and the UK’s traffic lights labelling system,^33^ which is worrying because labelling affects food choice and may explain the misalignment of UK national diet with government recommendations.^55,56^

High fat, salt, and sugar is the term for unhealthy products according to the FSA NPM. A consumer may believe that avoiding this category would ensure that they avoid all unhealthy products.^57^ Our study showed that the current FSA NPM 2004-05 failed to identify about 35-40% of processed and ultraprocessed products misaligned with UK dietary guidance. Even with the most conservative Eatwell Guide NPM (model No 1), >91% of processed and ultraprocessed products were identified as containing excess saturated fat, salt, or free sugar according to UK dietary guidance, but only 57% would be regulated as high fat, salt, and sugar according to the FSA NPM. Twenty years ago, a FSA working group discussed the importance of using recommended dietary intakes to design the healthy-less healthy threshold,^58,59^ but the final NPM created an even distribution of' less healthy, intermediate, and healthier choices.^58^ As a result, many products that misaligned with dietary guidance were exempt from regulation. The current FSA NPM 2004-05 does not reflect the latest UK recommendations for free sugar and fibre.^6^ A 2018 revision (which incorporated free sugar and fibre, but which has not yet been implemented) still identified 25-35% fewer processed and ultraprocessed products as unhealthy than the most conservative Eatwell Guide NPM (model No 1). The FSA NPM also assumes that favourable nutrients can offset unfavourable nutrients,^60^ but evidence for this assumption is lacking and the UK dietary recommendations also do not include this advice.^6^ Our study may have overestimated the number of products identified as unhealthy by the FSA NPM, because many food and drink categories are excluded from regulation, including savoury pastry products, such as sausage rolls, and chocolate based nut spreads.^11,61,62^ Consumers may be surprised to learn that a diet of non-high fat, salt, and sugar products can have higher salt, free sugar, and saturated fat content than the UK government recommends.

Excess energy based on UK dietary guidance as an individual parameter identified as unhealthy about 70-75% of all processed and ultraprocessed products. These findings are consistent with previous evidence indicating that processed and ultraprocessed products in the UK and elsewhere are energy dense and hyperpalatable,^33^ both mechanisms potentially driving excess consumption.^63^ The use of energy density as a criterion must carefully consider which items are in scope, to avoid potentially healthy food being unduly regulated (eg, minimally processed foods, such as unsalted nuts, would be captured with this criterion).

### Implications of this study

Our results showed that UK government guidance robustly captures unhealthy food, but also highlights the major inconsistencies between the current systems for identifying unhealthy food products in the UK, even compared with the most conservative NPM based on the Eatwell Guide.^44,45^ More than 90% of processed and ultraprocessed products are unhealthy according to current UK dietary guidance,^64^ which may explain why public adherence to this guidance is poor. Only 0.1% of individuals adhere to the nine Eatwell Guide recommendations,^65^ and average intakes of free sugar, saturated fat, and salt exceed recommendations^66,67^ while average intakes of fruit, vegetables, and fibre are below recommendations.^66,68,69^ Examples of products that change from not being unhealthy, according to the FSA NPM or the traffic lights labelling system, to being identified as unhealthy by the new Eatwell Guide NPMs, include ultraprocessed ready-to-eat or ready-to-heat meals, sandwiches, and fast food.

Improving UK public health requires a change in diets towards increased consumption of healthy minimally processed food. This change requires consistent, objective, and valid systems for identifying unhealthy and healthy products to help governments effectively regulate unhealthy food and promote healthy food. This study showed that current UK systems used in food policy (traffic lights labelling system and FSA NPM) are insufficient to support this change because the systems do not identify a significant proportion of products identified as unhealthy according to UK dietary guidance.

We do not propose a specific policy. Changes in definitions of unhealthy food products have far reaching consequences and require careful independent evaluation. This study has implications for policy because the findings highlight the large regulatory loopholes in, and incoherence between, the current systems, and the possible benefit for all stakeholders in having one system, with agreed thresholds aligned with UK dietary guidance, to identify unhealthy products for policy and regulation.

### Strengths and limitations of this study

The strengths of our study include the nationally representative food and drink supply for the UK with the most up-to-date food composition data. Also, the new NPMs were constructed based on well evidenced UK dietary guidance and were comparable with the FSA NPM and the traffic lights labelling system in terms of nutrients of consideration. A consistent database assessing processed and ultraprocessed products provided a sensitive approach to capture a representative selection of products to compare the relative performance of each NPM. The new Eatwell Guide NPMs in this analysis directly aligned with current best evidence for dietary guidance by applying diet level nutrient recommendations scaled proportionally to food and drink.

Limitations of our study include the use of the Nova classification. The Nova classification is widely used, but is not without limitations and criticisms.^22,23^ We used the Nova classification to identify processed and ultraprocessed products in a nationally representative food and drink database as a proxy for prepacked products that offered sensitivity over specificity, similar to previous food database analyses based on the FSA NPM,^32^ and to directly investigate untested claims that ultraprocessed products are already well described by existing legislation.^23–25^ We evaluated processed and ultraprocessed products with all of the NPMs to compare the relative performance of different nutrient profiling models with a representative product list. Hence this approach ensured consistency in comparing nutrient profiling models in identifying unhealthy products across the same number of items. Although this approach efficiently excluded exempt non-prepacked items, and is also the approach used by the Pan American Health Organization for identifying unhealthy items, the total number and range of products classified as unhealthy by the FSA NPM, as currently used in the real world, may be overestimated. We would expect minimal differences, however, in the relative proportion of items identified as unhealthy between the NPMs. This approach also offered consistency in investigating the second aim of assessing the proportion of ultraprocessed products captured by existing tools, as a subset of this product list. This approach resulted in minimally processed foods (MPF) not being tested in the analysis. Although including minimally processed foods may have been a useful reference of how well the NPMs discriminate between foods, and is a consideration for a future analysis, minimally processed foods were out of scope of the aims of this study.

The traffic lights labelling system can in theory be applied to any prepacked products, but because labelling is voluntary, many products that have excessive nutrients-to-limit are not labelled in the real world, but would be captured in our analysis. Furthermore, although assessing processed and ultraprocessed products provides a sensitive approach in identifying a representative selection of items to compare the relative performance of NPMs, this study does not propose this as the definitive list of items that should be regulated.

Prepacked items (ie, ultraprocessed and processed products) are, on average, more energy dense than minimally processed foods,^33^ and are often made by methods that increase energy density (removing water). Therefore, the application of the Eatwell Guide NPM that includes energy density would likely be expected to perform better than the traffic lights labelling system. NPM1 and NPM2, however, which do not assess energy density, were superior to the traffic lights labelling system and both FSA NPMs which consider energy density, although within a compensatory algorithm. The energy density component of NPM3 was from dietary guidelines from Food Standards Scotland and based on the Eatwell Guide and the dietary recommendations of the Scientific Advisory Committee on Nutrition, but is not widely implemented across the whole of the UK.

### Conclusions

Food and drink products with excess content of harmful nutrients, according to UK government dietary guidance, were not identified by current systems for regulating UK food and drink products. Our new NPMs, based on current UK government dietary guidance, identified as unhealthy significantly greater proportions of processed and ultraprocessed products than the FSA NPM and the front-of-pack traffic lights labelling system.

## Supplementary material

10.1136/bmjmed-2025-001750 online supplemental file 1

10.1136/bmjmed-2025-001750 online supplemental file 2

## Data Availability

Data are available upon reasonable request.

## References

[1] Afshin A, Sur PJ, Fay KA (2019). Health effects of dietary risks in 195 countries, 1990–2017: a systematic analysis for the Global Burden of Disease Study 2017. Lancet.

[2] Reitsma MB, Kendrick PJ, Ababneh E (2021). Spatial, temporal, and demographic patterns in prevalence of smoking tobacco use and attributable disease burden in 204 countries and territories, 1990–2019: a systematic analysis from the Global Burden of Disease Study 2019. Lancet.

[3] World Health Organisation (2018). Healthy diet. https://www.who.int/publications/m/item/healthy-diet-factsheet394.

[4] Belardo D, Michos ED, Blankstein R (2022). Practical, Evidence-Based Approaches to Nutritional Modifications to Reduce Atherosclerotic Cardiovascular Disease: An American Society For Preventive Cardiology Clinical Practice Statement. *Am J Prev Cardiol*.

[5] Gardner CD, Vadiveloo MK, Petersen KS (2023). Popular Dietary Patterns: Alignment With American Heart Association 2021 Dietary Guidance: A Scientific Statement From the American Heart Association. Circulation.

[6] NHS The Eatwell Guide. https://www.nhs.uk/live-well/eat-well/the-eatwell-guide/.

[7] Department of Health (2011). The nutrient profiling model.

[8] GOV.UK Front of Pack nutrition labelling guidance.

[9] Scientific Advisory Committee on Nutrition (SACN) Framework and methods for the evaluation of evidence that relates food and nutrients to health. 2023.. https://app.box.com/s/um0oo1p2wuw2xd15hp4us8dqe9u969ng.

[10] Cullum A (2024). Developing food-based dietary recommendations in the UK. Proc Nutr Soc.

[11] GOV.UK (2024). Restricting advertising of less healthy food or drink on TV and online: products in scope. https://www.gov.uk/government/publications/restricting-advertising-of-less-healthy-food-or-drink-on-tv-and-online-products-in-scope/restricting-advertising-of-less-healthy-food-or-drink-on-tv-and-online-products-in-scope.

[12] Northcott T, Lawrence M, Parker C (2023). Ecological regulation for healthy and sustainable food systems: responding to the global rise of ultra-processed foods. *Agric Hum Values*.

[13] Dicken SJ, Batterham RL (2022). The Role of Diet Quality in Mediating the Association between Ultra-Processed Food Intake, Obesity and Health-Related Outcomes: A Review of Prospective Cohort Studies. Nutrients.

[14] Monteiro CA, Cannon G, Levy R (2016). The star shines bright. World Nutr J.

[15] Monteiro CA, Cannon G, Moubarac J-C (2018). The UN Decade of Nutrition, the NOVA food classification and the trouble with ultra-processing. Public Health Nutr.

[16] Monteiro CA, Cannon G, Levy RB (2019). Ultra-processed foods: what they are and how to identify them. Public Health Nutr.

[17] World Health Organization, Food and Agriculture Organization of the United Nations, United Nations Children’s Fund (2024). Guidance for monitoring healthy diets globally.

[18] Food and Agriculture Organization of the United Nations, World Health Organization (2024). What are healthy diets? Joint statement by the Food and Agriculture Organization of the United Nations and the World Health Organization.

[19] Lichtenstein AH, Appel LJ, Vadiveloo M (2021). 2021 Dietary Guidance to Improve Cardiovascular Health: A Scientific Statement From the American Heart Association. Circulation.

[20] Koios D, Machado P, Lacy-Nichols J (2022). Representations of Ultra-Processed Foods: A Global Analysis of How Dietary Guidelines Refer to Levels of Food Processing. Int J Health Policy Manag.

[21] GOV.UK SACN statement on processed foods and health.

[22] Astrup A, Monteiro CA (2022). Does the concept of “ultra-processed foods” help inform dietary guidelines, beyond conventional classification systems? NO. *Am J Clin Nutr*.

[23] Parallel Parliament (2025). Debate: obesity: food and diet - 20th Jan 2025 - Andrew Gwynne extracts. https://www.parallelparliament.co.uk/mp/andrew-gwynne/debate/2025-01-20/commons/commons-chamber/obesity-food-and-diet.

[24] UK Parliament (2024). Written questions and answers - written questions, answers and statements. https://questions-statements.parliament.uk/written-questions/detail/2024-08-30/3616/.

[25] UK Parliament (2024). Processed Food - Written questions, answers and statements.

[26] Pan American Health Organization (2016). Pan American Health Organization nutrient profile model. https://www.who.int/publications/i/item/9789275118733.

[27] GOVUK (2020). NDNS: results from years 9 to 11 (2016 to 2017 and 2018 to 2019). https://www.gov.uk/government/statistics/ndns-results-from-years-9-to-11-2016-to-2017-and-2018-to-2019.

[28] Public Health England (2021). Evaluation of Change in Dietary Methodology in NDNS Rolling Programme: Stage 1..

[29] GOVUK (2021). Composition of foods integrated dataset (cofid). https://www.gov.uk/government/publications/composition-of-foods-integrated-dataset-cofid.

[30] MRC Human Nutrition Research. (2017). Food standards agency standard recipes database, 1992-2012. https://datacatalogue.ukdataservice.ac.uk/studies/study/8159#details.

[31] GOV.UK National diet and nutrition survey nutrient databank: pre-version 1 user guide.. https://datacatalogue.ukdataservice.ac.uk/studies/study/6533#details.

[32] Jenneson V, Greenwood DC, Clarke GP (2020). Restricting promotions of ‘less healthy’ foods and beverages by price and location: A big data application of UK Nutrient Profiling Models to a retail product dataset. Nutr Bull.

[33] Dicken SJ, Batterham RL, Brown A (2024). Nutrients or processing? An analysis of food and drink items from the UK National Diet and Nutrition Survey based on nutrient content, the NOVA classification and front of package traffic light labelling. Br J Nutr.

[34] Rauber F, Louzada ML da C, Martinez Steele E (2019). Ultra-processed foods and excessive free sugar intake in the UK: a nationally representative cross-sectional study. BMJ Open.

[35] GOV.UK Annex C: Basis of the Nutrient/Food Component and Scoring for the UK NPM 2004/5 and the Draft 2018 NPM. 2018..

[36] GOV.UK (2018). UK nutrient profiling model 2018 review. https://www.gov.uk/government/consultations/consultation-on-the-uk-nutrient-profiling-model-2018-review.

[37] GOVUK Nutrient profiling model 2018. https://www.gov.uk/government/publications/nutrient-profiling-model-2018.

[38] Scarborough P, Matthews A, Eyles H (2015). Reds are more important than greens: how UK supermarket shoppers use the different information on a traffic light nutrition label in a choice experiment. Int J Behav Nutr Phys Act.

[39] Balcombe K, Fraser I, Falco SD (2010). Traffic lights and food choice: A choice experiment examining the relationship between nutritional food labels and price. Food Policy.

[40] Dicken SJ, Batterham RL, Brown A (2025). Micronutrients or processing? An analysis of food and drink items from the UK National Diet and Nutrition Survey based on micronutrient content, the Nova classification and front-of-package traffic light labelling. Br J Nutr.

[41] Public Health England (2016). Government Recommendations for Energy and Nutrients for Males and Females Aged 1–18 Years and 19+ Years.

[42] Scientific Advisory, Committee on Nutrition (2003). Salt and Health.

[43] Scientific Advisory Committee on Nutrition (2007). Update on trans fatty acids and health: Position statement by the Scientific Advisory Committee on Nutrition.

[44] Klos B, Cook J, Crepaz L (2023). Impact of energy density on energy intake in children and adults: a systematic review and meta-analysis of randomized controlled trials. Eur J Nutr.

[45] Prentice AM, Jebb SA (2003). Fast foods, energy density and obesity: a possible mechanistic link. Obes Rev.

[46] Food Standards Scotland (2016). Scottish dietary goals.

[47] Tanner MA, Young MA (1985). Modeling Agreement among Raters. J Am Stat Assoc.

[48] Flexner N, Gomes FS, Mulligan C (2024). Comparing the Canadian front-of-pack labeling regulations with other mandatory approaches in the Americas and their ability to identify ultra-processed products. Public and Global Health.

[49] Canella DS, Pereira Montera V dos S, Oliveira N (2023). Food additives and PAHO’s nutrient profile model as contributors’ elements to the identification of ultra-processed food products. Sci Rep.

[50] Sacks G, Rayner M, Swinburn B (2009). Impact of front-of-pack “traffic-light” nutrition labelling on consumer food purchases in the UK. Health Promot Int.

[51] Jáuregui A, White CM, Vanderlee L (2022). Impact of front-of-pack labels on the perceived healthfulness of a sweetened fruit drink: a randomised experiment in five countries. Public Health Nutr.

[52] Roberto CA, Ng SW, Ganderats-Fuentes M (2021). The Influence of Front-of-Package Nutrition Labeling on Consumer Behavior and Product Reformulation. Annu Rev Nutr.

[53] Packer J, Russell SJ, Ridout D (2021). Assessing the Effectiveness of Front of Pack Labels: Findings from an Online Randomised-Controlled Experiment in a Representative British Sample. Nutrients.

[54] European Commission (2024). Food-based dietary guidelines in Europe. https://knowledge4policy.ec.europa.eu/health-promotion-knowledge-gateway/topic/food-based-dietary-guidelines-europe_en.

[55] Neale EP, Tapsell LC (2022). Nutrition and Health Claims: Consumer Use and Evolving Regulation. Curr Nutr Rep.

[56] Oostenbach LH, Slits E, Robinson E (2019). Systematic review of the impact of nutrition claims related to fat, sugar and energy content on food choices and energy intake. BMC Public Health.

[57] Arambepola C, Scarborough P, Rayner M (2008). Validating a nutrient profile model. Public Health Nutr.

[58] Scientific workshop to assess the food standards agency’s proposed approach to nutrient profiling. London. 2005.. https://webarchive.nationalarchives.gov.uk/ukgwa/20120415074730mp_/http://www.food.gov.uk/multimedia/pdfs/nutprofr.pdf.

[59] Rayner M, Scarborough P, Boxer A (2005). Nutrient profiles: development of final model: final report. https://webarchive.nationalarchives.gov.uk/ukgwa/20120415074730mp_/http://www.food.gov.uk/multimedia/pdfs/nutprofr.pdf.

[60] Department of Health (2011). Nutrient profiling technical guidance. https://www.gov.uk/government/publications/the-nutrient-profiling-model.

[61] British Retail Consortium (2023). HFSS Guidance: Products in out of scope.

[62] GS1 Q&A: Understanding the HFSS promotional location regulations: are you in scope.

[63] Fazzino TL, Courville AB, Guo J (2023). Ad libitum meal energy intake is positively influenced by energy density, eating rate and hyper-palatable food across four dietary patterns. *Nat Food*.

[64] Madruga M, Martínez Steele E, Reynolds C (2023). Trends in food consumption according to the degree of food processing among the UK population over 11 years. Br J Nutr.

[65] Scheelbeek P, Green R, Papier K (2020). Health impacts and environmental footprints of diets that meet the Eatwell Guide recommendations: analyses of multiple UK studies. BMJ Open.

[66] GOV.UK (2020). National diet and nutrition survey rolling programme years 9 to 11 (2016/2017 to 2018/2019).. https://www.gov.uk/government/statistics/ndns-results-from-years-9-to-11-2016-to-2017-and-2018-to-2019.

[67] Public Health England National diet and nutrition survey: assessment of salt intake from urinary sodium in adults (aged 19 to 64 years) in England, 2018 to 2019. https://www.gov.uk/government/statistics/national-diet-and-nutrition-survey-assessment-of-salt-intake-from-urinary-sodium-in-adults-aged-19-to-64-years-in-england-2018-to-2019.

[68] GOV.UK (2019). NDNS: time trend and income analyses for years 1 to 9. https://www.gov.uk/government/statistics/ndns-time-trend-and-income-analyses-for-years-1-to-9.

[69] Food Foundation (2025). The Broken Plate 2025.

[70] Buttriss JL (2016). The Eatwell Guide refreshed. Nutr Bull.

[71] GOVUK (2023). Restricting promotions of products high in fat, sugar or salt by location and by volume price: implementation guidance. https://www.gov.uk/government/publications/restricting-promotions-of-products-high-in-fat-sugar-or-salt-by-location-and-by-volume-price/restricting-promotions-of-products-high-in-fat-sugar-or-salt-by-location-and-by-volume-price-implementation-guidance.

[72] GOV.UK (2024). Less healthy food or drink: advertising and promotions restrictions. https://www.gov.uk/government/collections/less-healthy-food-or-drink-advertising-and-promotions-restrictions.

[73] European Union (2006). Regulation (EC) No 1924/2006 of the European Parliament and of the Council of 20 December 2006 on nutrition and health claims made on foods.

