# Supplementary material for: Evaluation of energy density and levels of fat, sugar, and salt in processed and ultra-processed food with existing and novel nutrient profiling models to identify unhealthy food and drink: cross sectional analysis of a nationally representative database of food and drink items
Source: BMJ Med. 2026 Nov 10;5(1):e001750. doi: 10.1136/bmjmed-2025-001750 (PMC13756371; doi:10.1136/bmjmed-2025-001750)
Supplement: Supplementary file 1 [file bmjmed-2025-001750.reviewer_comments.pdf]

PEER REVIEW HISTORY

BMJ Medicine publishes all reviews undertaken for accepted manuscripts. Reviewers are asked to complete a checklist review form and are provided with free text boxes to elaborate on their assessment. These free text comments are reproduced below.

ARTICLE DETAILS

**TITLE (PROVISIONAL)**	How much processed and ultra-processed food is energy dense and high in fat, sugar and salt? An evaluation of existing and novel nutrient profiling models in identifying unhealthy food and drink
**AUTHORS**	Dicken, Samuel; van Tulleken, Dolly; Brown, Adrian; Gomes, Fabio; van Tulleken, Christoffer

VERSION 1 - REVIEW

**REVIEWER NAME**	Barrett, Eden M.
**REVIEWER AFFILIATION**	University of New South Wales Sydney
**REVIEWER CONFLICT OF INTEREST**	None
**AI DISCLOSURE**	No
**DATE REVIEW RETURNED**	31-Jul-2025

**GENERAL COMMENTS**	Thank you for the opportunity to review this manuscript. This is an interesting paper that highlights that the current thresholds used in UK policy could be surely strengthened to improve differentiation between unhealthful and healthful foods, showing that only about half of UPF are considered unhealthy according to the FSA-NPM and traffic lights (defined as at least one red light). However, some choices in methods were not clear to me and I have some additional suggestions that I believe will enhance the clarity of the paper and the comparisons between models.<br><br>Major comments:<br>1. Can the authors clarify why the FSA-NPM was applied to all foods (within both Table 3s) and not to the 13 specific categories. That may have been a more useful and accurate comparison. It is mentioned as a loophole of the system in line 502 but this wasn't actually demonstrated by this study. Further, the application to all categories is mentioned as a limitation at line 517 but again, it wasn't clear why this was done.<br>2. Relatedly, the scope of the FSA-NPM is defined as 13 specific categories where products are generally PP or UPF (as per line 245), but then the second table 3 has tested it against minimally processed and culinary ingredients for unknown reasons.<br>3. It is not clear to me if identifying such a high proportion of 'processed' food i.e. Nova category 3 as unhealthy is necessarily desirable, because my understanding is that most of the poor health outcomes seen in relation to processing has focused on ultra-processed foods. Would it be reasonable for a processed food such as tinned salmon to be classified as unhealthy based on fat or energy content? What would be very helpful for context here is if the authors could include some example products that would shift from being allowed to be healthy as per the FSA-NPM or receive no red warning labels to being unhealthy according to the new NPMs, for both UPF and PP categories.

	Minor comments:<br>There are two Table 3.<br>Abstract line 44 missing the word “classified”<br>Methods line 184 missing the word “consumed” I think.<br>Methods line 224. In regards to items with missing data, line 204 says there is no missing data, so this confused me.<br>Second table 3 Energy row. Should this be <125 kcal?<br>Second table 3. FSA-NPM does not consider total fat.<br>Line 287-295. Logic a bit hard to follow because the FSA-NPM considers energy content too.

**REVIEWER NAME**	Brand-Miller, Jennie
**REVIEWER AFFILIATION**	University of Sydney CAR, Charles Perkins Centre
**REVIEWER CONFLICT OF INTEREST**	None
**AI DISCLOSURE**	No
**DATE REVIEW RETURNED**	05-Aug-2025

**GENERAL COMMENTS**	Flagging “healthy” foods in the supermarket is something many people want to see. However, the devil is in the detail. The goal posts keep getting moved. This paper shows that the most recent healthy food guidelines put >95% of processed foods outside the goal posts. Previous algorithms were far too generous, capturing less than half of all “unhealthy” foods.<br>Although the authors fail to discuss it, it appears to be irrelevant whether they are processed or ultra-processed (UPF). Yet, UPFs are the latest dietary demon: not only made in a food factory, they contain food additives that make them ultra-palatable. The analysis suggests that high energy density (kcal/g) is the problem, rather than the food additives per se. The authors believe the current situation is seriously wrong. Food companies (especially industrial giants) are able to trick the public into buying foods that are unhealthy according to updated guidelines. Unfortunately, if updated criteria are used, fewer than 1 in 20 factory products will make the grade.<br><br>Strengths<br>• A good overview of current nutrient profiling systems used in the UK<br>• Inconsistencies among them are listed<br>• Separate analysis of processed vs ultraprocessed foods<br>• Coders of PP or UPF were blinded to nutrient content (not to ingredients)<br>• Nationally representative nutrient and food database<br><br><br>Weaknesses<br>• The NOVA classification is highly controversial and ambiguous<br>• No comparison of processed products (PP) vs UPF<br>• Eatwell Guide may not be ‘best’ just because it’s the most recent<br>• The Eatwell Guides are not significantly different to each other (discussion)<br>• No rationale for free sugars at <5%E vs saturated fat at <10%E?<br><br>Suggestions for authors<br>1. Clearly, I’m not fond of the NOVA classification. The authors have uncritically accepted it as useful, but don’t justify their viewpoint,

	rather taking it as face value. A more critical approach would be valuable.<br>2. Figure 1. I would like to see PP and UPF compared with each other. In my mind, there's no point to using the NOVA classification if there's no difference between PP and UPF. Without food processing, we would all starve.<br>3. The key attribute of UPF is food additives. Demonising these, many of them being nature-identical, without due cause risks yet another nutrition "fiasco". To my knowledge all food additives in the UK have been tested according to stringent criteria and proven to be safe at levels that are normally encountered in food. Is that correct?<br>4. Surely Grandma also made foods that were energy dense and far too palatable? Why not develop a simple metric that takes both E density and palatability into account?

**REVIEWER NAME**	Perera, Rafael
**REVIEWER AFFILIATION**	University of Oxford, Primary Care Health Sciences
**REVIEWER CONFLICT OF INTEREST**	None
**AI DISCLOSURE**	I have used Grammarly to help me with spelling and grammar. Aiming to enhance the utility of the review.
**DATE REVIEW RETURNED**	25-Sep-2025

**GENERAL COMMENTS**	Stats Review:<br><br>The current manuscript provides information on the performance of three novel nutrient profiling models (NPMs) based on Eatwell guidelines when compared to the Food Standards Agency-NPM (two types: 2004/5 and 2018) and the Traffic Lights system in identifying 'unhealthy foods' as determined by the NOVA classification of processed (PP) and ultra-processed (UP) foods. They find that the new guidelines identify a substantially higher number of PP and UP as unhealthy (over 90%). In contrast, the FSA -NPMs and the Traffic Lights system only identify between half and two-thirds of the PP and UP foods as unhealthy.<br><br>This is an interesting paper, highlighting important discrepancies between the different systems and aims to create some clarity by proposing the new NPMs as a way to link nutrient profile models with the NOVA classification. The study can be viewed as a classification problem, similar to a diagnostic accuracy question. In this case, the reference standard is the NOVA classification, with several potential index tests (all the NPMS, both old and new) being explored to determine which is best.<br><br>Based on the above premise, the first critical aspect to be justified or at least explored is the suitability of the NOVA classification as a reference standard. A cursory examination of this field suggests that this issue has not been completely resolved and may not be entirely feasible, given the complexities of nutritional epidemiology. Given this, some basic exploration, either in the Background or Discussion section, is needed to discuss the advantages and limitations of using this classification as the reference standard. This would allow readers to better understand the current state of the lack of consensus.<br><br>Regarding the assessment of the different NPMs, the current analysis is partial and biased. The current approach assumes that

	NOVA classified PP and UP foods are all unhealthy. This means that minimally processed food and processed culinary ingredients are, by inference, considered healthy. Given this, an adequate evaluation of the different NPMs would require the inclusion of healthy AND unhealthy foods. In the context of a diagnostic accuracy study, this would mean the inclusion of non-cases and cases. The current evaluation of only unhealthy foods for the new NPMs is equivalent to analysing only cases, and aiming to optimise sensitivity. This is achieved by the naïve classifier, which labels all products tested as 'unhealthy', thereby achieving 100% sensitivity. This is why, in a diagnostic accuracy study, the controls are also tested against the index test to explore its performance in discriminating between the two groups. Only by understanding the ability of the newly proposed NPMs to distinguish between healthy and unhealthy foods can we determine if they are better than the current ones. For this, I would suggest employing some discrimination statistic.<br><br>As a side note, the use of Wald statistics suggests that a 'global test' has been used to determine if 'at least one' of the NPMs is different to the others. There is good separation in the performance, as summarised by the 95%CI, but there does not seem to be a direct comparison between NPMs in the traditional sense. Related to this point, please provide the code used for the analysis so this can be replicated if required.

VERSION 1 – AUTHOR RESPONSE

Reviewer: 1
Dr. Eden M. Barrett, University of New South Wales Sydney
Comments to the Author

Thank you for the opportunity to review this manuscript. This is an interesting paper that highlights that the current thresholds used in UK policy could be surely strengthened to improve differentiation between unhealthful and healthful foods, showing that only about half of UPF are considered unhealthy according to the FSA-NPM and traffic lights (defined as at least one red light). However, some choices in methods were not clear to me and I have some additional suggestions that I believe will enhance the clarity of the paper and the comparisons between models.

Thank you for the kind words on our manuscript, and for the constructive comments, which we have addressed below.

Major comments:

1. Can the authors clarify why the FSA-NPM was applied to all foods (within both Table 3s) and not to the 13 specific categories. That may have been a more useful and accurate comparison. It is mentioned as a loophole of the system in line 502 but this wasn't actually demonstrated by this study. Further, the application to all categories is mentioned as a limitation at line 517 but again, it wasn't clear why this was done.

Thank you for this comment.

The aim of this study was to compare the 'relative' performance of different NPMs in identifying unhealthy food, therefore a representative sample of relevant products needed to be identified to do so.

The FSA-NPM is largely applied to prepacked items, with the majority of exempt items being 'non-prepacked food and drink' along with some specific prepacked exemptions. https://www.gov.uk/government/publications/restricting-promotions-of-products-high-in-fat-sugar-or-salt-by-location-and-by-volume-price/restricting-promotions-of-products-high-in-fat-sugar-or-salt-by-location-and-by-volume-price-implementation-guidance?utm_source=chatgpt.com#what-food-is-in-scope

Within the UK NDNS food and drink database, there is no direct method to identify the relevant items or even categories within scope for the FSA-NPM, according to the 13 specific categories in the UK NDNS database (as they do not map onto the predefined UK NDNS food/drink subgroups, and many items in the database are not explicitly listed as found in the in-scope guidance). Given the complexity and difficulty in attempting to align the categories and items of FSA-NPM with the UK NDNS database, the approach taken to opt for sensitivity over specificity was in alignment with the approach of Jenneson et al., who noted similar difficulties in applying the FSA-NPM 2004 and FSA-NPM 2018 to a commercial database (myfood24):

***"This approach prioritised sensitivity over specificity; inclusion of some out of scope products is tolerated to minimise the exclusion of in-scope products."* https://onlinelibrary.wiley.com/doi/full/10.1111/nbu.12468**

In this regard, we used PP and UPP as defined by the Nova classification as a proxy for prepacked items that efficiently excluded exempt non-prepacked items, and is also the approach used by PAHO for identifying unhealthy items. The relevance to PAHO is clarified in the Introduction in lines 321-322 and Methods in lines 754-755 *("by using the PAHO/WHO approach of proportionally scaling diet-level intake goals to apply thresholds to products[22]")*. Whilst this study did not apply the FSA-NPM to the 13 specific categories, the application to PP and UPP aligns closely with in-scope items. For example, a number of specific exclusions from within the 13 in-scope categories would also be excluded already by focussing on PF/UPF, such as raw nuts and seeds from Category 2, natural yoghurt from Category 10, or plain potatoes from Category 12.

Furthermore, the main aim was to compare the 'relative' performance of different NPMs, and therefore this approach ensures consistency in comparing NPMs in identifying unhealthy products across a broader range of products but the same number of items (e.g. to capture items such as Nutella or Kellogg's CocoPops that would be generally considered high in sugar). As such, it would be expected that there would be minimal difference in the relative proportion of items identified as unhealthy between NPMs. This approach also offered consistency in addressing our second aim of assessing the proportion of UPP captured by existing tools, as a subset of this selection.

We have provided further clarification of this approach in the Methods:

Lines 603-607:

"There is no publicly available database of all existing food and drink in the UK, nor a database of products in-scope for regulation by FSA-NPM.[23] Therefore, a nationally representative database of all food and drink in the UK was used to identify PP and UPP according to the Nova classification[33] as a proxy for prepacked items in the UK (and similar to PAHO[22]), and for identifying UPP."

And, we acknowledge the potential differences of this in the Discussion:

Line 1578-1581:

"Notably, our study may overestimate the number of products captured as unhealthy by the FSA-NPM, since many food and drink categories are excluded from regulation, including savoury pastry products like sausage rolls, or chocolate-based nut spreads.(46,68,69)"

And limitations:

Lines 1697-1727:

"Limitations include the use of the Nova classification. Whilst the Nova classification is widely used, it is not without limitations and criticism39,40. It was used here to identify PP and UPP in a nationally representative food and drink database as a proxy for prepacked products that offered sensitivity over specificity, similar to previous food database analyses using FSA-NPM48, and to directly address untested claims that UPP are already well described by existing legislation40–42. We subjected total PP and UPP to all NPMs in order to compare the relative performance of different NPMs to a representative product list, and therefore this approach ensured consistency in comparing NPMs in identifying unhealthy products across the same number of items. However, whilst this approach efficiently excluded exempt non-prepacked items, and is also the approach used by PAHO for identifying unhealthy items, it may overestimate the total number and range of products classified as unhealthy by the FSA-NPM as currently used in the real world. However, it would be expected that there would be minimal difference in the relative proportion of items identified as unhealthy between NPMs. This approach also offered consistency in addressing the second aim of assessing the proportion of UPP captured by existing tools, as a subset of this product list. In addition, Traffic Lights can in theory be applied to any prepacked products, but since they are voluntary, there are many products excessive in nutrients-to-limit to which they are not applied in the real world, which would be captured in this analysis. Furthermore, whilst assessing PP and UPP provides a sensitive approach to capture a representative selection of items to compare the relative performance of NPMs, this study does not propose that this would be the exact list of items to be subject to regulation. Prepacked items (i.e. UPP and PP) are on average are more energy dense than MPF49, and are often made by methods that increase energy density (removing water), Therefore, it may be expected that the application of the Eatwell NPM that includes energy density would likely be expected to perform better than Traffic Lights. However, both Eatwell-NPM-1 and Eatwell-NPM-2 which do not assess energy density were superior to Traffic Lights and both FSA-NPMs which consider energy density, although within a compensatory algorithm. Whilst the energy density component of Eatwell-NPM-3 is from FSS dietary guidelines and based on Eatwell and SACN dietary recommendations, it is not widely implemented across the whole of the UK."

2. Relatedly, the scope of the FSA-NPM is defined as 13 specific categories where products are generally PP or UPF (as per line 245), but then the second table 3 has tested it against minimally processed and culinary ingredients for unknown reasons.

Thank you for this comment.

In relation to the comment above and to other peer reviewers, we have clarified the use of PP and UPP as the core dataset for consideration: as combination of a proxy for in-scope items for the FSA-NPMs, to address the question of whether UPP is covered by existing policy tools in the UK, and to align with the PAHO approach of in-scope items when proportionally scaling diet-

level intake goals to apply thresholds to products. As such, all NPMs are tested against PP and UPP only. In alignment with the study aims and to ensure consistency with using PP and UPP only across each NPMs, the additional MPF and PCI comparisons have been removed for clarity.

Table 3 refers to scope of food and drink subject to the application of the NPM. The top row of Table 3 has been amended to specify the scope of items in this analysis.

A footnote has been added to Table 3 (now Table 4) to outline the scope of items applicable:

Line 1169-1170:

"Scope of food and drink subject to the application of all NPMs are all processed and/or ultra-processed products"

This has also been further addressed in the Methods section in lines 702-706:

"All PP and UPP were used as products in scope, as it is not possible to directly map in-scope categories of the FSA-NPM to the food and drink groups of the NDNS database, or directly identify specific exempt items from within scope categories. This approach to prioritise sensitivity over specificity for capturing items for consideration is similar to previous approaches applying FSA-NPM to nutrient databases.[48]"

3. It is not clear to me if identifying such a high proportion of 'processed' food i.e. Nova category 3 as unhealthy is necessarily desirable, because my understanding is that most of the poor health outcomes seen in relation to processing has focused on ultra-processed foods. Would it be reasonable for a processed food such as tinned salmon to be classified as unhealthy based on fat or energy content? What would be very helpful for context here is if the authors could include some example products that would shift from being allowed to be healthy as per the FSA-NPM or receive no red warning labels to being unhealthy according to the new NPMs, for both UPF and PP categories.

Thank you for this comment.

We appreciate this comment and it highlights important considerations to be given to the Eatwell NPMs when making policy proposals for food regulation. Importantly, the aim of this analysis was to compare the relative performance of the NPMs to capture unhealthy products from a database of industrially produced prepacked products. As in our response above to the first major comment, PP was included as part of a sensitive proxy approach to identify a representative set of prepacked products from the nationally representative database, and to be in alignment with the PAHO approach. We are not proposing that this would be the exact list of items that could be subject to regulation (and such a discussion is beyond the scope of this paper), but as noted above, this provided a sensitive approach to capture a representative selection of items similar to FSA-NPM in-scope items and PAHO to compare the relative performance of NPMs. We have then simply and objectively applied NPMs based on UK dietary guidelines to identify how many products would or not meet that criteria.

We have updated the discussion to make it clear that we simply report on how products that are identified as unhealthy according to Traffic Lights or FSA-NPMs, compared with products that are identified as unhealthy using Eatwell NPMs:

Lines 1612-1617:

“This paper does not make a specific policy proposal. Changes in definitions of unhealthy food have far reaching consequences and require careful independent evaluation. This study does have implications for policy as it highlights the large regulatory loopholes in, and incoherence between current systems, and the possible benefit for all stakeholders in having a single system using agreed thresholds aligned with UK dietary guidance to identify unhealthy products for policy and regulation.”

And further clarified this distinction in the limitations:

Line 1715-1718:

“Furthermore, whilst assessing PP and UPP provides a sensitive approach to capture a representative selection of items to compare the relative performance of NPMs, this study does not propose that this would be the exact list of items to be subject to regulation.”

Of course, not all tinned salmon are necessarily equal in terms of their ingredients and nutritional composition. Tinned salmon with added sodium may or may not be considered excessive in sodium depending on the final composition. In terms of examples of products that would shift from not being less healthy as per the FSA-NPM or receive no red warning labels in Traffic Lights, to being identified as unhealthy according to the new Eatwell NPMs, these include mostly ultra-processed ready-to-eat or ready-to-heat meals, sandwiches and fast food, such as chicken wraps, macaroni cheese, fisherman's pie, toddler meals.

We have now included this detail in the Discussion:

Line 1616-1619:

“Examples of products that shift from not being unhealthy according to FSA-NPM or Traffic Lights to being identified as unhealthy according to the new Eatwell NPMs include ultra-processed ready-to-eat or ready-to-heat meals, sandwiches and fast food.”

Minor comments:
There are two Table 3.

Thank you for this highlighting this discrepancy. The table numbers have now been amended.

Abstract line 44 missing the word “classified”

Thank you for identifying the missing word.

Methods line 184 missing the word “consumed” I think.

Thank you for this suggestion. This analysis utilised the nutrient database from NDNS, rather than of the items explicitly consumed by participants. However, the items indeed reflect food and drink consumed as the reason for their inclusion in the national database.

We have updated the sentence with this:

Line 599-600:

"This was a cross-sectional analysis of a nationally representative database of food and drink items consumed in the UK."

Methods line 224. In regards to items with missing data, line 204 says there is no missing data, so this confused me.

Thank you for this comment and for highlighting the statement. Upon checking the list of items, the 7 items with missing data in the nutrient database for one or more of the nutrients required across all NPMs were already 7 of the 16 items described earlier with no corresponding item in the NDNS nutrient databank. Therefore, this sentence has been removed for clarity as it is redundant (after exclusion of the 16 items mentioned above, there are no additional observations with missing data for nutrients).

We have clarified this by amending line 728-733:

"Out of 3,105 total items in the database, 109 items were designated as outside the Nova classification (e.g., fish oil supplements and multivitamins), and 16 items had no corresponding item in the NDNS nutrient databank as previously reported.[49] Alcoholic drinks were excluded from this analysis as they are subject to other specific regulations (n = 35). Out of the 2,945 remaining items, 1,636 items were classified as UPP, 262 were PP, 61 were processed culinary ingredients and 986 were minimally processed foods."

Second table 3 Energy row. Should this be <125 kcal?

Thank you for spotting this typo. We have amended this to ≤ 125 kcal in Table 2 as this is the recommended target (and >125 kcal in Table 3).

Second table 3. FSA-NPM does not consider total fat.

Thank you for spotting the typo in Table 4, this has now been removed.

Line 287-295. Logic a bit hard to follow because the FSA-NPM considers energy content too.

Thank you for highlighting this.

We have updated the section for clarity:

Lines 762-764:

"The first NPM was based on thresholds for saturated fat, free sugar, and sodium (Eatwell-NPM-1) to align with unfavourable nutrients (excluding energy) used in FSA-NPM whilst accounting for current UK dietary guidance focussing on free sugars, not total sugars."

Reviewer: 2
Prof. Jennie Brand-Miller, University of Sydney CAR
Comments to the Author

Flagging "healthy" foods in the supermarket is something many people want to see. However, the devil is in the detail. The goal posts keep getting moved. This paper shows that the most recent healthy food guidelines put >95% of processed foods outside the goal posts. Previous algorithms were far too generous, capturing less than half of all "unhealthy" foods.

Although the authors fail to discuss it, it appears to be irrelevant whether they are processed or ultra-processed (UPF). Yet, UPFs are the latest dietary demon: not only made in a food factory, they contain food additives that make them ultra-palatable. The analysis suggests that high energy density (kcal/g) is the problem, rather than the food additives per se. The authors believe the current situation is seriously wrong. Food companies (especially industrial giants) are able to trick the public into buying foods that are unhealthy according to updated guidelines. Unfortunately, if updated criteria are used, fewer than 1 in 20 factory products will make the grade.

Strengths
• A good overview of current nutrient profiling systems used in the UK
• Inconsistencies among them are listed
• Separate analysis of processed vs ultraprocessed foods
• Coders of PP or UPF were blinded to nutrient content (not to ingredients)
• Nationally representative nutrient and food database

We thank the reviewer for acknowledging the strengths of this manuscript.

Weaknesses
• The NOVA classification is highly controversial and ambiguous
• No comparison of processed products (PP) vs UPF
• Eatwell Guide may not be 'best' just because it's the most recent
• The Eatwell Guides are not significantly different to each other (discussion)
• No rationale for free sugars at <5%E vs saturated fat at <10%E?

Suggestions for authors

1. Clearly, I'm not fond of the NOVA classification. The authors have uncritically accepted it as useful, but don't justify their viewpoint, rather taking it as face value. A more critical approach would be valuable.

Thank you for this comment.

We apologise if there has been miscommunication in our writing. We did not intend to uncritically accept the Nova classification as useful, or assume that all UPF is unhealthy in this

manuscript. A key reason for assessing the Nova classification in this study is outlined in the Introduction, that members of the UK government have stated that UPP are already well described by existing legislation, including HFSS according to FSA-NPM, or UK dietary guidance, but this statement is largely untested. Therefore, we aimed to test the assertion made by UK public health regulators in a hypothesis-blind way: how much UPF is considered to be unhealthy according to UK dietary guidance and other current tools? We see that according to the Eatwell Guide, almost all is considered 'unhealthy' (99%), but around two thirds of these according to FSA-NPM and traffic lights. Our observation is also not to suggest that food companies are tricking consumers, but simply acknowledging the discrepancies between UK dietary guidance and the regulatory tools to help consumers adhere to these guidelines.

The second key reason why the Nova classification was used, was because there is no publicly available database of all existing food and drink in the UK, or even a database of industrially prepacked products in-scope for regulation by FSA-NPM[22]. Therefore, we used PP and UPP according to the Nova classification[32] as a proxy for prepacked items in the UK. This also aligned with the approach used by PAHO. We have outlined this in further detail in lines 603-607:

"There is no publicly available database of all existing food and drink in the UK, nor a database of products in-scope for regulation by FSA-NPM.[23] Therefore, a nationally representative database of all food and drink in the UK was used to identify PP and UPP according to the Nova classification[33] as a proxy for prepacked items in the UK (and similar to PAHO[22]), and for identifying UPP."

To further clarify this, we have also added content in the Introduction, outlining that Nova has been criticised, and that we explicitly wanted to see how much of it was misaligned with dietary guidance:

Lines 579-585:

"However, UK dietary guidance does not formally advise on the purpose and extent of industrial food processing,[38] and does not feature in the FSA-NPM or Traffic Lights. The Nova classification is controversial and has been criticised for being too ambiguous, difficult to implement and offering limited value in addition to existing regulatory tools.[39] For example, members of the UK government have stated that the definition of UPP is too broad for policy use, and that UPP are already well described by existing legislation, including HFSS according to FSA-NPM, or UK dietary guidance.[40–42]"

Furthermore, to clarify this focus on the proportion of PP and UPP misaligned with dietary guidance, we provided reference to the proportion of PP and UPP captured as unhealthy relative to the Eatwell NPMs in the Discussion:

Line 1492-1494:

"This is consistent with our finding that the Traffic Lights NPM identified only half the number of PP and UPP as unhealthy compared to NPMs based on UK dietary guidance."

Line 1500-1502:

"When comparing Eatwell-NPM-2 and Traffic Lights (like-for-like NPMs based on excess fat, saturated fat, sugar and salt), Eatwell-NPM-2 identified nearly twice as many PP and UPP as unhealthy."

Line 1512-1513:

"Our study shows that the current FSA-NPM-2004/5 fails to identify around 40-45% of PP and UPP misaligned with UK dietary guidance."

We have acknowledged the use of Nova classification and the rationale for its use in the limitations:

Lines 1697-1707*:*

"Limitations include the use of the Nova classification. Whilst the Nova classification is widely used, it is not without limitations and criticism[39,40]. It was used here to identify PP and UPP in a nationally representative food and drink database as a proxy for prepacked products that offered sensitivity over specificity, similar to previous food database analyses using FSA-NPM[48], and to directly address untested claims that UPP are already well described by existing legislation[40–42]. We subjected total PP and UPP to all NPMs in order to compare the relative performance of different NPMs to a representative product list, and therefore this approach ensured consistency in comparing NPMs in identifying unhealthy products across the same number of items."

We hope that these additional justifications and explanations on the approach clarify matters.

2. Figure 1. I would like to see PP and UPF compared with each other. In my mind, there's no point to using the NOVA classification if there's no difference between PP and UPF. Without food processing, we would all starve.

Thank you for this comment.

As noted above, a key reason why the Nova classification was used was because there is no publicly available database of all existing food and drink in the UK, or even a database of industrially prepacked products in-scope for regulation by FSA-NPM[22]. Therefore, we used PP and UPP according to the Nova classification[32] as a proxy for prepacked items in the UK. This also aligned with the approach used by PAHO. We have outlined this in further detail in lines 603-607.

We agree that without food processing, we would all starve, and also agree that this is an important comparison to make between PP and UPP. Therefore, we have provided this comparison in the proportion of PP and UPP identified as unhealthy in Table 4 with use of the confidence intervals, and a comparison across each excessive nutrients-to-limit in Table 5. When Traffic Lights and FSA-NPMs are used to identify unhealthy products, a non-significantly greater proportion of UPP than PP are identified as unhealthy and/or excessive in one or more nutrients-to-limit. Whereas when dietary guidelines based Eatwell-NPM criteria are used, the proportions of PP and UPP identified as unhealthy/containing excessive amounts of nutrients-to-limit are more similar. However, it is important to note that we are assessing food product composition, and not actual intakes or health outcomes resulting from or associated with this intake. These comparisons on health risks associated with PP and UPP intake and their non-nutrient profile and composition have been conducted by other studies, but are not possible using the dataset analysed here. Of relevance in our previous study using the same database, we observed differences in macronutrient and micronutrient content between UPF and PF per

100 g and per 100 kcal: https://pmc.ncbi.nlm.nih.gov/articles/PMC11043912/ and https://pmc.ncbi.nlm.nih.gov/articles/PMC11946030/ .

Lastly, this study is not proposing to use the Nova classification in policy or regulation. As mentioned in the above comment, the aim of this study was to test the assertion made by UK public health regulators in a hypothesis-blind way: how much UPP is considered to be unhealthy according to UK dietary guidance and other current tools.

3. The key attribute of UPF is food additives. Demonising these, many of them being nature-identical, without due cause risks yet another nutrition "fiasco". To my knowledge all food additives in the UK have been tested according to stringent criteria and proven to be safe at levels that are normally encountered in food. Is that correct?

Thank you for this comment.

Whilst the operational definition to identify UPF rests on the presence of ingredients of sole industrial use, or cosmetic additives, food additives are not the key attribute per se. For example, MPF, PCI and PF can all contain additives with functional purposes (e.g. antioxidants, microorganism proliferation, or to ensure durability: Supplementary Table 1: https://static-content.springer.com/esm/art%3A10.1038%2Fs41574-025-01143-7/MediaObjects/41574_2025_1143_MOESM1_ESM.pdf). The Nova classification therefore does not threaten food safety or durability. In this regard in the Nova classification, cosmetic additives serve as a practical marker to identify products undergoing the UPF business model and purpose of processing to create highly profitable products.

Our analysis also makes no suggestion to demonise additives, nor specifically any attempt to regulate them, and the downstream policy implications of our study here focusses on nutrients and energy density, and does not demonise food additives. As mentioned the aim of the study was to test the assertion made by UK public health regulators in a hypothesis-blind way: how much UPF is considered to be unhealthy according to UK dietary guidance and other current tools, as stated previously.

Lines 1612-1617:

"This paper does not make a specific policy proposal. Changes in definitions of unhealthy food have far reaching consequences and require careful independent evaluation. This study does have implications for policy as it highlights the large regulatory loopholes in, and incoherence between current systems, and the possible benefit for all stakeholders in having a single system using agreed thresholds aligned with UK dietary guidance to identify unhealthy products for policy and regulation."

We hope these additions clarify matters.

4. Surely Grandma also made foods that were energy dense and far too palatable? Why not develop a simple metric that takes both E density and palatability into account?

Thank you for this comment. Whilst development of novel metrics based on palatability would be an interesting separate analysis to consider, the aim of this study was to compare existing metrics used across policy, rather than to generate a completely novel metric to provide alignment with current tools and have a single aligned metric with the Eatwell Guide (as outlined in the Discussion). This is therefore beyond the scope of this analysis.

We thank the reviewer for their supportive comments on the value of our manuscript. To summarise our response to the Reviewer's concerns, the Nova classification was used as a tool to identify industrially prepacked foods due to the lack of such a database in the UK. We have added critical points to the manuscript to outline its use, as well as providing further detail to the limitations.

Reviewer: 3
Prof. Rafael Perera, University of Oxford
Comments to the Author

bmjmed-2025-001750: Is the classification of unhealthy food in the UK fit for purpose? An evaluation of existing and novel nutrient profiling models in identifying unhealthy food and drink according to UK dietary guidance.

Stats Review:

The current manuscript provides information on the performance of three novel nutrient profiling models (NPMs) based on Eatwell guidelines when compared to the Food Standards Agency-NPM (two types: 2004/5 and 2018) and the Traffic Lights system in identifying 'unhealthy foods' as determined by the NOVA classification of processed (PP) and ultra-processed (UP) foods. They find that the new guidelines identify a substantially higher number of PP and UP as unhealthy (over 90%). In contrast, the FSA-NPMs and the Traffic Lights system only identify between half and two-thirds of the PP and UP foods as unhealthy.

This is an interesting paper, highlighting important discrepancies between the different systems and aims to create some clarity by proposing the new NPMs as a way to link nutrient profile models with the NOVA classification. The study can be viewed as a classification problem, similar to a diagnostic accuracy question. In this case, the reference standard is the NOVA classification, with several potential index tests (all the NPMS, both old and new) being explored to determine which is best.

We thank the reviewer for their supportive comments on the value of our manuscript.

Based on the above premise, the first critical aspect to be justified or at least explored is the suitability of the NOVA classification as a reference standard. A cursory examination of this field suggests that this issue has not been completely resolved and may not be entirely feasible, given the complexities of nutritional epidemiology. Given this, some basic exploration, either in the Background or Discussion section, is needed to discuss the advantages and limitations of using this classification as the reference standard. This would allow readers to better understand the current state of the lack of consensus.

Thank you for this comment to further clarify the use of the Nova classification.

The use of the Nova classification was two-fold:

First, to identify a proxy set of prepacked products to compare the relative performance of NPMs, and second, due to the debate and criticism around the Nova classification:

The FSA-NPM is largely applied to prepacked items, with the majority of exempt items being 'non-prepacked food and drink' along with some specific prepacked exemptions. https://www.gov.uk/government/publications/restricting-promotions-of-products-high-in-fat-sugar-or-salt-by-location-and-by-volume-price/restricting-promotions-of-products-high-in-fat-sugar-or-salt-by-location-and-by-volume-price-implementation-guidance?utm_source=chatgpt.com#what-food-is-in-scope

Within the UK NDNS food and drink database, there is no direct method to identify the relevant items or even categories within scope for the FSA-NPM, according to the 13 specific categories in the UK NDNS database (as they do not map onto the predefined UK NDNS food/drink subgroups, and many items in the database are not explicitly listed as found in the in-scope guidance). Given the complexity and difficulty in attempting to align the categories and items of FSA-NPM with the UK NDNS database, the approach taken to opt for sensitivity over specificity was in alignment with the approach of Jenneson et al., who noted similar difficulties in applying the FSA-NPM 2004 and FSA-NPM 2018 to a commercial database (myfood24):

***"This approach prioritised sensitivity over specificity; inclusion of some out of scope products is tolerated to minimise the exclusion of in-scope products."* https://onlinelibrary.wiley.com/doi/full/10.1111/nbu.12468**

In this regard, we used PP and UPP as defined by the Nova classification as a proxy for prepacked items that efficiently excluded exempt non-prepacked items, and is also the approach used by PAHO for identifying unhealthy items. The relevance to PAHO is clarified in the Introduction in lines 321-322 and Methods in lines 754-755 *("by using the PAHO/WHO approach of proportionally scaling diet-level intake goals to apply thresholds to products[22]")*. Whilst this study did not apply the FSA-NPM to the 13 specific categories, the application to PP and UPP aligns closely with in-scope items. For example, a number of specific exclusions from within the 13 in-scope categories would also be excluded already by focussing on PF/UPF, such as raw nuts and seeds from Category 2, natural yoghurt from Category 10, or plain potatoes from Category 12.

Furthermore, the main aim was to compare the 'relative' performance of different NPMs, and therefore this approach ensures consistency in comparing NPMs in identifying unhealthy products across a broader range of products but the same number of items (e.g. to capture items such as Nutella or Kellogg's CocoPops that would be generally considered high in sugar). As such, it would be expected that there would be minimal difference in the relative proportion of items identified as unhealthy between NPMs. This approach also offered consistency in addressing our second aim of assessing the proportion of UPP captured by existing tools, as a subset of this selection.

We have provided further clarification of this approach in the Methods:

Lines 603-607:

"There is no publicly available database of all existing food and drink in the UK, nor a database of products in-scope for regulation by FSA-NPM.[23] Therefore, a nationally representative database of all food and drink in the UK was used to identify PP and UPP according to the Nova classification[33] as a proxy for prepacked items in the UK (and similar to PAHO[22]), and for identifying UPP."

And, we acknowledge the potential differences of this in the Discussion:

Line 1578-1581:

"Notably, our study may overestimate the number of products captured as unhealthy by the FSA-NPM, since many food and drink categories are excluded from regulation, including savoury pastry products like sausage rolls, or chocolate-based nut spreads.(46,68,69)"

And limitations:

Lines 1697-1702*:*

"Limitations include the use of the Nova classification. Whilst the Nova classification is widely used, it is not without limitations and criticism39,40. It was used here to identify PP and UPP in a nationally representative food and drink database as a proxy for prepacked products that offered sensitivity over specificity, similar to previous food database analyses using FSA-NPM48, and to directly address untested claims that UPP are already well described by existing legislation40–42."

Regarding the assessment of the different NPMs, the current analysis is partial and biased. The current approach assumes that NOVA classified PP and UP foods are all unhealthy. This means that minimally processed food and processed culinary ingredients are, by inference, considered healthy. Given this, an adequate evaluation of the different NPMs would require the inclusion of healthy AND unhealthy foods. In the context of a diagnostic accuracy study, this would mean the inclusion of non-cases and cases. The current evaluation of only unhealthy foods for the new NPMs is equivalent to analysing only cases, and aiming to optimise sensitivity. This is achieved by the naïve classifier, which labels all products tested as 'unhealthy', thereby achieving 100% sensitivity. This is why, in a diagnostic accuracy study, the controls are also tested against the index test to explore its performance in discriminating between the two groups. Only by understanding the ability of the newly proposed NPMs to distinguish between healthy and unhealthy foods can we determine if they are better than the current ones. For this, I would suggest employing some discrimination statistic.

Thank you for this comment.

We apologise if there has been miscommunication in our writing. We did not intend to give the impression that we assume that PP and UPP defined by the Nova classification are all inherently unhealthy. As mentioned, an aim of the study was to test the assertion made by UK public health regulators in a hypothesis-blind way: to evaluate how much UPP is considered to be unhealthy according to UK dietary guidance and other current tools, as stated in the previous response. Importantly, the current approach used here does not make any assumption that PP and UPP identified using the Nova classification are all unhealthy.

The other aim of this study was to compare the 'relative' performance of different NPMs, in identifying unhealthy food. Therefore a representative sample of relevant products needed to be identified. We therefore used UPP and PP in our analysis for several reasons: as a proxy for in-scope items for FSA-NPMs as no such publicly available database exists, to address the question of whether UPP are covered by existing tools, and to align with PAHO approach. So, for consistency, all NPMs are tested against PP and UPP only. (The top row of Table 3 has now been adjusted to clarify this scope of items for consideration in this study).

We then make two main distinct and complementary analyses.

The first aim relates to how well aligned existing policy tools are in identifying unhealthy items compared with UK dietary guidance. This analysis is outlined in Table 4.

The results are stratified by PP and UPP (Table 4), showing and comparing the proportion of products that would be identified as unhealthy according to NPMs based on the Eatwell Guide, and according to FSA and Traffic Lights. In this study, we do not describe the Nova classification as an NPM, nor imply that 100% of UPP and PP should be defined as unhealthy by it. Instead, we compared whether the proportions of products identified as unhealthy and not unhealthy by the six NPMs (not including the Nova classification as a criterion) differed between them in a pairwise fashion. Although the proportions of PP and UPP that are defined as not unhealthy are not explicitly shown in the table (they are implicitly present as 1 minus the proportions shown), we have tested whether the vectors of proportions of cases (unhealthy according to each NPM) and non-cases (not unhealthy according to each NPM) differ using Wald statistics for homogeneity with Rao-Scott first- and second-order corrections. The non-cases then refer to the PP and/or UPP that were not defined as unhealthy by each NPM.

The methods section has been amended to help clarify this:

Lines 1041-1044:

"To compare the performance of NPMs in identifying PP and UPP as unhealthy and not unhealthy, the proportions of PP and UPP identified as unhealthy and not unhealthy by each NPM was compared against all other NPMs using Wald statistics for homogeneity, applying Rao-Scott first- and second-order corrections."

The second aim relates to whether UPP are captured by existing policy tools. This is outlined in Table 6. The rationale for this is described in the Introduction:

Lines 583-586:

"…members of the UK government have stated that the definition of UPP is too broad for policy use, and that UPP are already well described by existing legislation, including HFSS according to FSA-NPM, or UK dietary guidance.[40–42] No study to date has directly tested this claim."

As this question does relate to UPP specifically, this analysis does consider that there is a reference standard for establishing which products are UPP, regardless of the controversies and debate about whether these should be the focus of policy and regulation or not. However, these controversies are now explicitly mentioned in the Introduction:

Line 581-583:

"The Nova classification is controversial and has been criticised for being too ambiguous, difficult to implement and offering limited value in addition to existing regulatory tools.[39] For example…"

UPP have been most commonly defined by the Nova classification. Therefore, we used Nova to define UPP in the dataset to provide evidence to respond the question on how much UPP would be identified as unhealthy according to UK dietary guidance and other current NPMs. This again is not assuming that UPP are inherently unhealthy.

We then assessed and compared the proportion of UPP that would be identified as unhealthy and not unhealthy according to the six NPMs in Table 6. In this case, the reference is UPP. This reference does not define any product as unhealthy or not unhealthy, it simply states all products in the subset are UPP to answer the question. Then, agreement statistics comparing each NPM against the reference are presented, as well as pairwise comparisons of the

magnitude of differences between the proportions of UPP identified as unhealthy (and implicitly the proportions identified as not unhealthy) between all possible pairs of NPMs.

To conduct the proposed suggestion for a discrimination analysis to assess the ability of each NPM to distinguish between unhealthy and not unhealthy products requires a gold-standard reference NPM that truly defines which products are unhealthy and which are not. We could assume that Eatwell-3 NPM serves this purpose as the gold-standard reference and best available discriminator of unhealthy and not unhealthy products, given that it does include the most comprehensive set of parameters established by UK dietary guidelines. However, as there is no consensus agreement on the best NPM for this purpose, we feel this analysis would not be possible at the moment without introducing additional assumptions. Also, discriminant analyses are usually useful when researchers are interested in finding variables that can predict the grouping of observations, where statistics and data help drive the decision on the clusters. Our objective was to test how existing NPMs and dietary guidelines-based NPMs identify products as unhealthy and not unhealthy, and not to define a criterion based on the composition of products in the dataset and discriminant statistics grouping these.

We have therefore further clarified the current analytical approach in the Limitations:

Lines 1697-1727:

"Limitations include the use of the Nova classification. Whilst the Nova classification is widely used, it is not without limitations and criticism39,40. It was used here to identify PP and UPP in a nationally representative food and drink database as a proxy for prepacked products that offered sensitivity over specificity, similar to previous food database analyses using FSA-NPM48, and to directly address untested claims that UPP are already well described by existing legislation40–42. We subjected total PP and UPP to all NPMs in order to compare the relative performance of different NPMs to a representative product list, and therefore this approach ensured consistency in comparing NPMs in identifying unhealthy products across the same number of items. However, whilst this approach efficiently excluded exempt non-prepacked items, and is also the approach used by PAHO for identifying unhealthy items, it may overestimate the total number and range of products classified as unhealthy by the FSA-NPM as currently used in the real world. However, it would be expected that there would be minimal difference in the relative proportion of items identified as unhealthy between NPMs. This approach also offered consistency in addressing the second aim of assessing the proportion of UPP captured by existing tools, as a subset of this product list. In addition, Traffic Lights can in theory be applied to any prepacked products, but since they are voluntary, there are many products excessive in nutrients-to-limit to which they are not applied in the real world, which would be captured in this analysis. Furthermore, whilst assessing PP and UPP provides a sensitive approach to capture a representative selection of items to compare the relative performance of NPMs, this study does not propose that this would be the exact list of items to be subject to regulation."

Combined, we hope that these clarifications on the analyses help to explain our analytical approach.

Note: In relation to other comments by peer reviewers, we have clarified the use of PP and UPP as the core dataset for consideration: as combination of a proxy for in-scope items for the FSA-NPMs, to address the question of whether UPP is covered by existing policy tools in the UK, and to align with the PAHO approach of in-scope items when proportionally scaling diet-level intake goals to apply thresholds to products. As such, all NPMs are tested against PP and UPP only.

In alignment with the study aims and to ensure consistency with using PP and UPP only across each NPMs, the additional MPF and PCI comparisons have been removed for clarity.

As a side note, the use of Wald statistics suggests that a 'global test' has been used to determine if 'at least one' of the NPMs is different to the others. There is good separation in the performance, as summarised by the 95%CI, but there does not seem to be a direct comparison between NPMs in the traditional sense. Related to this point, please provide the code used for the analysis so this can be replicated if required.

Thank you for this comment.

We have amended the text in the Methods section to clarify that the Wald statistics for homogeneity with Rao-Scott first- and second-order corrections has been applied to test the difference between all possible pairs of NPMs compared.

Lines 1041-1044:

"To compare the performance of NPMs in identifying PP and UPP as unhealthy and not unhealthy, the proportions of PP and UPP identified as unhealthy and not unhealthy by each NPM was compared against all other NPMs using Wald statistics for homogeneity, applying Rao-Scott first- and second-order corrections."

We are more than happy to provide the code used for the analysis, and have added a statement:

Lines 1852-1853:

"Code Availability

The code used to conduct analyses can be made available upon request to the authors."

We thank the reviewer for allowing us the opportunity to strengthen our manuscript by incorporating additional details on data and analyses, and addressing these methodological concerns into the manuscript to enhance its reliability and interpretability. We have added critical points to the manuscript to outline its use, as well as providing further detail to the limitations.

VERSION 2 – REVIEW

**REVIEWER NAME**	Brand-Miller, Jennie
**REVIEWER AFFILIATION**	University of Sydney CAR, Charles Perkins Centre
**REVIEWER CONFLICT OF INTEREST**	None
**AI DISCLOSURE**	No
**DATE REVIEW RETURNED**	06-Mar-2026

**GENERAL COMMENTS**	I thank the authors for addressing my questions thoughtfully. There is a great deal of assumed 'local' (country-specific) knowledge behind the original paper. Although the revised paper is improved, I think it could be made easier to read and understand.<br>Suggestions<br>1. The title of the paper is a question (Is the classification of unhealthy food fit for purpose?). In their responses, the authors make it clear that a specific question they want to address is 'How much ultraprocessed food is unhealthy according to official UK dietary guidance? In my opinion, this question as the title would make the whole article clearer.<br>2. The authors say they have used the Nova classification as a reference for identifying prepackaged foods because there is no alternative. This seems like obfuscation to me, given the stated aim. Why not be upfront?<br>3. Have the authors answered their own question directly? If current UK guidance (EatWell) identifies 99 out of 100 UPF, surely it's fit-for-purpose? Is the UK government right – the UPF are already covered? Are the other NPM not fit-for-purpose? Or is the Nova classification demonising all processed food? In my opinion, it's the latter. And it could have unintended consequences. I think the 4th reviewer is correct is suggesting that there is inherient bias in the methodology. This could be a stated limitation.<br>4. The fact that a high proportion of PP are also unhealthy by current criteria suggests that the 'diagnostic criteria' are too broad. The authors point out that 75% of foods fail on E-density alone. All nuts are E-dense yet clearly healthy foods. Could the authors suggest that E-density should not be a criterion. Could this be a point in the Discussion?<br>5. All the acronyms are a mouthful, if not mind-boggling. Could they be simplified, even NPM#1, #2 and 3# etc after first identification? Re-state during Discussion.

**REVIEWER NAME**	Perera, Rafael
**REVIEWER AFFILIATION**	University of Oxford, Primary Care Health Sciences
**REVIEWER CONFLICT OF INTEREST**	None
**AI DISCLOSURE**	I have used Grammarly to help with spelling and grammar.
**DATE REVIEW RETURNED**	04-Mar-2026

**GENERAL COMMENTS**	Stats Review:<br>After reading this new version of the manuscript and the authors' responses to the editor's and reviewers' comments, I believe the authors have adequately addressed them, and the manuscript is suitable for publication in BMJ Medicine.<br><br>There is one minor point that I would like the authors to consider, which might slightly affect their presentation and potentially also the analyses used. The authors highlight that the novel NPMs they present are intended to illustrate a gap between what the FSA-NPM(s) and the Traffic Lights classify as HFS and what the UK government dietary guidance (Eatwell) currently recommends. I believe they also state that the newly created NPMs are not meant to replace recommendations, as this is too complex to achieve with the current approach. Given this, I suggest that most of their discussion should use the most conservative estimates based on

	Eatwell-NPM-1 instead of Eatwell-NPM-3. This will highlight that even in this most conservative case, there are significant gaps.<br><br>Using the same logic, I am unsure that the Ranking of agreement with the NOVA classifier is useful for the paper's objectives. The intention is not to say which NPM is ‘best’ but to highlight the current gap with the Eatwell guidance. I think this analysis could be removed from the main paper and included as supplementary material.<br><br>My take is that these two adjustments would prevent the manuscript from being criticised for prescribing new NPMs, while this (in my understanding) is not the purpose of the work.

VERSION 2 – AUTHOR RESPONSE

Reviewer: 3

Prof. Rafael Perera, University of Oxford

Comments to the Author

bmjmed-2025-001750.R1: Is the classification of unhealthy food in the UK fit for purpose? An evaluation of existing and novel nutrient profiling models in identifying unhealthy food and drink according to UK dietary guidance.

Stats Review:

After reading this new version of the manuscript and the authors' responses to the editor’s and reviewers’ comments, I believe the authors have adequately addressed them, and the manuscript is suitable for publication in BMJ Medicine.

Thank you for your praise regarding the strength of the updated manuscript.

There is one minor point that I would like the authors to consider, which might slightly affect their presentation and potentially also the analyses used. The authors highlight that the novel NPMs they present are intended to illustrate a gap between what the FSA-NPM(s) and the Traffic Lights classify as HFS and what the UK government dietary guidance (Eatwell) currently recommends. I believe they also state that the newly created NPMs are not meant to replace recommendations, as this is too complex to achieve with the current approach. Given this, I suggest that most of their discussion should use the most conservative estimates based on Eatwell-NPM-1 instead of Eatwell-NPM-3. This will highlight that even in this most conservative case, there are significant gaps.

Thank you for this suggestion.

We have now focussed the discussion on the conservative estimates based on Eatwell-NPM-1 instead of Eatwell-NPM-3 (now NPM#1 and NPM#3 following Reviewer 2's comments below to simplify the NPM names).

Lines 771-772:

"NPM#1, the most conservative NPM, identified over 91% of PP and UPP as unhealthy and misaligned with UK dietary guidance."

In the next paragraph discussing the results for Traffic Lights, we have now added the comparison to the conservative NPM#1, and removed the comparison with NPM#2:

Lines 791-792:

"NPM#1, the most conservative NPM, identified 43-46% more (nearly twice as many) PP and UPP as unhealthy than Traffic Lights."

In the third paragraph, which discusses the results for FSA-NPM, we have focussed the comparison on the most conservative NPM#1:

Lines 830-834:

"Our study shows that the current FSA-NPM-2004/5 fails to identify around 35-40% of PP and UPP misaligned with UK dietary guidance. Even with the most conservative Eatwell Guide NPM (NPM#1), over 91% of PP and UPP were identified as containing excess saturated fat, salt and/or free sugar according to UK dietary guidance, but only 57% would be subject to HFSS regulation."

Lines 838-841:

"The currently used FSA-NPM-2004/5 does not reflect latest UK recommendations for free sugar and fibre.[6] A 2018 revision (which reflected these, but which has not yet been implemented) still identified 25-35% fewer PP and UPP compared to the most conservative Eatwell Guide NPM (NPM#1)."

This has also been amended in the Implications section of the Discussion:

Lines 871-874:

"These results show that UK government guidance robustly captures unhealthy food, but also highlights the significant inconsistencies between current systems identifying unhealthy food in the UK, even when comparing them to the most conservative NPM based on the Eatwell Guide.[55,56] Over 90% of PP and UPP are unhealthy according to current UK dietary guidance,[75]"

We thank the Reviewer for this useful suggestion, which highlights as the Reviewer points out, that even in this most conservative case, there are significant gaps between Traffic Lights and FSA-NPM, and UK dietary guidance.

Using the same logic, I am unsure that the Ranking of agreement with the NOVA classifier is useful for the paper's objectives. The intention is not to say which NPM is 'best' but to highlight the current gap with the Eatwell guidance. I think this analysis could be removed from the main paper and included as supplementary material.

Thank you for this helpful suggestion.

To ensure clarity of the main findings which highlight the gap between existing regulatory tools and Eatwell Guidance to identify unhealthy food and drink, we have now moved the Ranking of agreement results in Table 6 into the Supplementary Materials, as Supplementary Table 4.

We have also moved most of the Results section discussing the ranking of agreement analysis into the Supplementary Materials as well (Lines 649-657 in original manuscript), to aid with clarity.

My take is that these two adjustments would prevent the manuscript from being criticised for prescribing new NPMs, while this (in my understanding) is not the purpose of the work.

We thank the Reviewer for suggesting these two adjustments, both of which we have made, allowing us to strengthen our manuscript.

Reviewer: 2

Prof. Jennie Brand-Miller, University of Sydney CAR

Comments to the Author

I thank the authors for addressing my questions thoughtfully. There is a great deal of assumed 'local' (country-specific) knowledge behind the original paper. Although the revised paper is improved, I think it could be made easier to read and understand.

Suggestions

1. The title of the paper is a question (Is the classification of unhealthy food fit for purpose?). In their responses, the authors make it clear that a specific question they want to address is 'How much ultraprocessed food is unhealthy according to official UK dietary guidance? In my opinion, this question as the title would make the whole article clearer.

Thank you for this comment and suggestion of a new title.

As stated in the Introduction, our three aims were:

(1) to calculate the proportion of PP and UPP in the UK identified as unhealthy according to the current FSA-NPM and Traffic Lights.

(2) to develop novel NPMs based on UK dietary recommendations in the Eatwell Guide.

(3) to compare these proportions with the proportion of PP and UPP identified as unhealthy according to the current FSA-NPM and Traffic Lights with the proportion of PP and UPP identified as unhealthy according to the novel NPMs based on the Eatwell Guide.

To sufficiently capture these three aims of this study, including the question suggested by the Reviewer, we agree therefore that the Title could be re-written. We have therefore re-written the title as:

Lines 1-3:

"How much processed and ultra-processed food is energy dense and high in fat, sugar and salt? An evaluation of existing and novel nutrient profiling models in identifying unhealthy food and drink"

2. The authors say they have used the Nova classification as a reference for identifying prepackaged foods because there is no alternative. This seems like obfuscation to me, given the stated aim. Why not be upfront?

Thank you for this comment.

We apologise if this comment was not sufficiently addressed in the previous round of review.

Indeed, one of the study aims was to assess how much UPP is captured by existing UK tools to identify unhealthy food. This was highlighted in the introduction, and was due to comments made by members of UK government:

Lines 395-401:

"The Nova classification is controversial and has been criticised for being too ambiguous, difficult to implement and offering limited value in addition to existing regulatory tools.[39] For example, members of the UK government have stated that the definition of UPP is too broad for policy use, and that UPP are already well described by existing legislation, including HFSS according to FSA-NPM, or UK dietary guidance.[40–42] No study to date has directly tested this claim."

However, this was not the sole aim of the study and there were other key aims that were also addressed. This in particular was comparing the performance of each of the NPMs. As such, our three aims were:

(1) to calculate the proportion of PP and UPP in the UK identified as unhealthy according to the current FSA-NPM and Traffic Lights

(2) to develop novel NPMs based on UK dietary recommendations in the Eatwell Guide

(3) to compare these proportions with the proportion of PP and UPP identified as unhealthy according to the current FSA-NPM and Traffic Lights with the proportion of PP and UPP identified as unhealthy according to the novel NPMs based on the Eatwell Guide.

This is highlighted in the Introduction:

Lines 289-294:

"…products rated as unhealthy by one system can be rated as not unhealthy by another, and vice versa. It is possible that many products deemed unhealthy according to UK government dietary guidance are not captured by the other two systems, and thus may remain unregulated and heavily marketed, and could be perceived as healthy by consumers. However, no study to date has directly tested this."

And indeed therefore, PP and UPP defined by the Nova classification served as the reference dataset of prepackaged foods to consistently and simultaneously address these questions. We apologise if this seemed like obfuscation. We have further clarified this reference in the Methods:

Lines 422-450:

"There is no publicly available database of all existing food and drink in the UK, nor a database of products in-scope for regulation by FSA-NPM.[23] Therefore, a nationally representative database of all food and drink in the UK was used to identify PP and UPP according to the Nova classification[33] as a proxy for prepacked items in the UK (and similar to PAHO[22]), and for identifying the proportion of PP and UPP as unhealthy by NPMs."

The updated Title that focuses on both PP and UPP, not just UPP, also helps to emphasise that the Nova classification is being used as a proxy for prepackaged items, this is also reflected in the Abstract such as in the Abstract Conclusion:

Lines 92-97:

"Food and drink products that, according to UK government dietary guidance, contain excess amounts of harmful nutrients are not identified by current systems for regulating UK food and drink (FSA-NPM and Traffic Lights). The most conservative novel NPM based on current UK government dietary guidance identified significantly greater proportions of PP and UPP as unhealthy compared with FSA-NPM and Traffic Lights, and 91-92% of all PP and UPP."

3. Have the authors answered their own question directly? If current UK guidance (EatWell) identifies 99 out of 100 UPF, surely it's fit-for-purpose? Is the UK government right – the UPF are already covered? Are the other NPM not fit-for-purpose? Or is the Nova classification demonising all processed food? In my opinion, it's the latter. And it could have unintended consequences. I think the 4th reviewer is correct is suggesting that there is inherent bias in the methodology. This could be a stated limitation.

Thank you for this suggestion.

We absolutely agree that UK dietary guidance is fit for purpose, it is the comparison of Traffic Lights and FSA-NPM with UK dietary guidance that is under consideration in this study.

This is outlined in the Abstract Conclusion:

Lines 92-99:

“Food and drink products that, according to UK government dietary guidance, contain excess amounts of harmful nutrients are not identified by current systems for regulating UK food and drink (FSA-NPM and Traffic Lights). The most conservative novel NPM based on current UK government dietary guidance identified significantly greater proportions of PP and UPP as unhealthy compared with FSA-NPM and Traffic Lights, and 91-92% of all PP and UPP. These findings have important implications for food policy and the appropriateness of UK dietary guidance to identify unhealthy products.”

This has been outlined in the Summary section:

Line 151-155:

“Section 3: How might these results change the focus of research or clinical practice?

This study highlights regulatory loopholes and misalignment of FSA-NPM and front of pack ‘Traffic Light’ labels with UK Dietary Guidance in identifying unhealthy food and drink, and the possible benefit for all stakeholders in having a single system using agreed thresholds aligned with UK dietary guidance to identify unhealthy products for policy and regulation.”

And in the Discussion:

Lines 884-890:

“Improving UK public health requires a shift in diets towards increased consumption of healthy minimally processed food. This requires consistent, objective and valid systems for identifying unhealthy and healthy products to help governments effectively regulate unhealthy food and promote healthy food. However, this study shows that current UK systems used in food policy (Traffic Lights and FSA-NPM) are insufficient to support this as they do not identify a significant proportion of products identified as unhealthy according to UK dietary guidance.”

We are in no way suggesting in this paper to use the Nova classification in regulatory policy in the UK, but instead we suggest that regulatory tools (FSA-NPM and Traffic Lights) are not sufficiently capturing unhealthy foods as compared with UK dietary guidance. This is outlined in the Implications section of the Discussion:

Lines 892-906:

“This paper does not make a specific policy proposal. Changes in definitions of unhealthy food have far reaching consequences and require careful independent evaluation. This study does have implications for policy as it highlights the large regulatory loopholes in, and incoherence between current systems, and the possible benefit for all stakeholders in having a single system using agreed thresholds aligned with UK dietary guidance to identify unhealthy products for policy and regulation.”

Furthermore, we believe that your useful suggestion to change the Title of the manuscript has been helpful in framing that the aims of this study are to determine how much unhealthy food is captured by

different systems used in UK, and not trying to make a judgement call on any policy proposals, or suggesting any use of the Nova classification.

We hope these additions and clarifications sufficiently answer your concerns.

4. The fact that a high proportion of PP are also unhealthy by current criteria suggests that the 'diagnostic criteria' are too broad. The authors point out that 75% of foods fail on E-density alone. All nuts are E-dense yet clearly healthy foods. Could the authors suggest that E-density should not be a criterion. Could this be a point in the Discussion?

Thank you for this comment.

We appreciate that this is an important point, which we have added to the discussion.

Line 854-867:

"The use of energy density as a criterion must carefully consider which items are in-scope, to avoid potentially healthy food being unduly regulated, e.g. minimally processed foods such as unsalted nuts would be captured with this criterion."

It is worth noting that raw nuts would not be considered PP or UPP – salted nuts would be considered PP, and would 'fail' based on salt content as well. This is what the FSA-NPM considers with its in-scope items, by only applying to categories of pre-packed items, and not all minimally processed foods (and hence why we used a reference dataset as close as possible to this with a similar product range to conduct the analysis in the study as described in the Methods), such that minimally processed foods like raw nuts would not be discriminated.

5. All the acronyms are a mouthful, if not mind-boggling. Could they be simplified, even NPM#1, #2 and 3# etc after first identification? Re-state during Discussion.

Thank you for highlighting this and offering the suggestion to improve clarity. We have now renamed Eatwell-NPM-1 as NPM#1, Eatwell-NPM-2 as NPM#2 and Eatwell-NPM-3 as NPM#3 throughout the manuscript, to improve readability.

At the beginning of the Discussion, we have also re-stated what each of these NPMs capture, to make it easier for readers to follow:

Lines 766-770:

"This is the first study to compare the performance and consistency of existing UK food systems for classification of unhealthy food, and three novel NPMs based on UK dietary guidance (NPM#1 identifying excess saturated fat, free sugar, and/or sodium; NPM#2 identifying excess saturated fat, free sugar, sodium and/or total fat, and NPM#3 identifying excess saturated fat, free sugar, sodium, total fat, and/or energy density)."

We thank the Reviewer for their constructive comments and opportunity to improve the clarity and interpretation of the analysis.
