# Supplementary material for: Evaluation of energy density and levels of fat, sugar, and salt in processed and ultra-processed food with existing and novel nutrient profiling models to identify unhealthy food and drink: cross sectional analysis of a nationally representative database of food and drink items
Source: BMJ Med. 2026 Nov 10;5(1):e001750. doi: 10.1136/bmjmed-2025-001750 (PMC13756371; doi:10.1136/bmjmed-2025-001750)
Supplement: Supplementary file 3 [file bmjmed-5-1-s002.pdf]

STROBE Statement—checklist of items that should be included in reports of observational studies

	Item No.	Recommendation	Page No.	Relevant text from manuscript
**Title and abstract**	1	(*a*) Indicate the study's design with a commonly used term in the title or the abstract	1	"How much processed and ultra-processed food is energy dense and high in fat, sugar and salt? An evaluation of existing and novel nutrient profiling models in identifying unhealthy food and drink"
		(*b*) Provide in the abstract an informative and balanced summary of what was done and what was found	2	"Objectives: To assess the proportions of processed (PP) and ultra-processed products (UPP) in the UK that are high in fat, salt, sugar (HFSS) and energy density captured by the two definitions used to regulate unhealthy food products in the UK – the Food Standards Agency nutrient profile model, a formula which designates products as 'less healthy' or not (FSA-NPM), and front of pack 'Traffic Light' labels, and to compare these proportions to the proportions of PP and UPP identified by UK dietary guidance using novel NPMs."
**Introduction**				
Background/rationale	2	Explain the scientific background and rationale for the investigation being reported	5-9	"In 2017, poor diet overtook tobacco as the leading global cause of premature mortality…"
Objectives	3	State specific objectives, including any prespecified hypotheses	9	"Therefore, the aims of this study were.."
**Methods**				
Study design	4	Present key elements of study design early in the paper	9-10	"This is a cross-sectional analysis of a nationally representative database of food and drink items in the UK."
Setting	5	Describe the setting, locations, and relevant dates, including periods of recruitment, exposure, follow-up, and data collection	10-11	"There is no publicly available database of all existing food and drink in the UK. Therefore, a nationally representative database of all food and drink in the UK was used to identify PP and UPP according to the Nova classification…"
Participants	6	(*a*) *Cohort study*—Give the eligibility criteria, and the sources and methods of selection of participants. Describe methods of follow-up	10-11	Nutrient composition data of PP and UPP from Year 12 of the National Diet and Nutrition Survey (NDNS) were used. NDNS

		Case-control study—Give the eligibility criteria, and the sources and methods of case ascertainment and control selection. Give the rationale for the choice of cases and controls<br>*Cross-sectional study*—Give the eligibility criteria, and the sources and methods of selection of participants		is a repeated cross-sectional survey that collects dietary intake from a nationally representative sample of the UK population, aged 1.5 years and older and living in private households, since 2008[38]. Until 2019 (Years 1-11), dietary assessment in NDNS was conducted using four-day food diaries across consecutive days. In 2019/20 (Year 12), diaries were replaced with four non-consecutive, multiple-pass, 24-hour dietary recalls using Intake24 (https://intake24.co.uk), with an updated food and drink database to reflect the change in diet assessment[39]. The rationale and subsequent updates to the food and drink database are reported elsewhere[39].
		(*b*) *Cohort study*—For matched studies, give matching criteria and number of exposed and unexposed<br>*Case-control study*—For matched studies, give matching criteria and the number of controls per case		
Variables	7	Clearly define all outcomes, exposures, predictors, potential confounders, and effect modifiers. Give diagnostic criteria, if applicable	12-15, Table 3	"The proportion of PP and UPP identified as unhealthy were calculated according to the FSA-NPM algorithms, the Traffic Light system, and three novel NPMs based on the Eatwell Guide."
Data sources/ measurement	8*	For each variable of interest, give sources of data and details of methods of assessment (measurement). Describe comparability of assessment methods if there is more than one group	12-15, Table 3	"The FSA-NPM is a binary system to classify food and drink as 'healthy' or 'less healthy'…"
Bias	9	Describe any efforts to address potential sources of bias	18	"Categorical variables were described using counts and percentages. The proportion of PP and UPP identified as unhealthy according to all NPMs were reported with 95% confidence intervals (95%CI). The proportion of PP and UPP identified as unhealthy were then reported according to each nutrient or energy threshold in Traffic Lights and Eatwell

				NPMs (FSA-NPM is an overall score to identify unhealthy products and is therefore not reported).<br><br>To compare the performance of NPMs in identifying PP and UPP as unhealthy and not unhealthy, the proportions of PP and UPP identified as unhealthy and not unhealthy by each NPM was compared against all other NPMs using Wald statistics for homogeneity, applying Rao-Scott first- and second-order corrections.<br><br>To quantify the performance of NPMs in identifying unhealthy UPP, prevalence ratios (PR) with 95%CI were used to compare the magnitude difference in the proportion of UPP identified as unhealthy by each NPM.<br><br>To assess the overlap between each NPM and the Nova classification of UPP, Cohen's kappa with 95%CI was used to compare the agreement between the proportion of UPP identified as unhealthy by each NPM and the total number of UPP. Non-homogeneous log-linear models were then used to rank each NPM according to their level of agreement with the Nova classification of UPP.[55]"
Study size	10	Explain how the study size was arrived at	11	Nutrient composition data of PP and UPP from Year 12 of the National Diet and Nutrition Survey (NDNS) were used. NDNS is a repeated cross-sectional survey that collects dietary intake from a nationally representative sample of the UK population, aged 1.5 years and older and living in private households, since 2008[38]. Until 2019 (Years 1-11), dietary assessment in NDNS was conducted using four-day food diaries across consecutive days. In 2019/20 (Year 12), diaries were replaced with four non-consecutive, multiple-pass, 24-hour dietary recalls using

		Intake24 (https://intake24.co.uk), with an updated food and drink database to reflect the change in diet assessment[39]. The rationale and subsequent updates to the food and drink database are reported elsewhere[39].

Continued on next page

Quantitative variables	11	Explain how quantitative variables were handled in the analyses. If applicable, describe which groupings were chosen and why	14	"Categorical variables were described using counts and percentages. The proportion of PP and UPP identified as unhealthy according to all NPMs were reported with 95% confidence intervals (95%CI). The proportion of PP and UPP identified as unhealthy were then reported according to each nutrient or energy threshold in Traffic Lights and Eatwell NPMs (FSA-NPM is an overall score to identify unhealthy products and is therefore not reported)."
Statistical methods	12	(*a*) Describe all statistical methods, including those used to control for confounding	14	"Categorical variables were described using counts and percentages. The proportion of PP and UPP identified as unhealthy according to all NPMs were reported with 95% confidence intervals (95%CI). The proportion of PP and UPP identified as unhealthy were then reported according to each nutrient or energy threshold in Traffic Lights and Eatwell NPMs (FSA-NPM is an overall score to identify unhealthy products and is therefore not reported).<br><br>To compare the performance of NPMs in identifying PP and UPP as unhealthy and not unhealthy, the proportions of PP and UPP identified as unhealthy and not unhealthy by each NPM was compared against all other NPMs using Wald statistics for homogeneity, applying Rao-Scott first- and second-order corrections.<br><br>To quantify the performance of NPMs in identifying unhealthy UPP, prevalence ratios (PR) with 95%CI

				were used to compare the magnitude difference in the proportion of UPP identified as unhealthy by each NPM.<br><br>To assess the overlap between each NPM and the Nova classification of UPP, Cohen's kappa with 95%CI was used to compare the agreement between the proportion of UPP identified as unhealthy by each NPM and the total number of UPP. Non-homogeneous log-linear models were then used to rank each NPM according to their level of agreement with the Nova classification of UPP.[55]"
		(*b*) Describe any methods used to examine subgroups and interactions	N/A	
		(*c*) Explain how missing data were addressed	12	"16 items had no corresponding item in the NDNS nutrient databank as previously reported[43]…"
		(*d*) *Cohort study*—If applicable, explain how loss to follow-up was addressed<br>*Case-control study*—If applicable, explain how matching of cases and controls was addressed<br>*Cross-sectional study*—If applicable, describe analytical methods taking account of sampling strategy	N/A	
		(*e*) Describe any sensitivity analyses	N/A	
**Results**				
Participants	13*	(a) Report numbers of individuals at each stage of study—eg numbers potentially eligible, examined for eligibility, confirmed eligible, included in the study, completing follow-up, and analysed	12	"Out of 3,105 total items in the database, 109 items were designated as outside the Nova classification (e.g., fish oil supplements and multivitamins), and 16 items had no corresponding item in the NDNS nutrient databank as previously reported.[42] Alcoholic drinks were excluded from this analysis as they are subject to other specific regulations (n = 35)**.** [40] Out of the 2,945 remaining items, 1,636

				items were classified as UPP, 262 were PP, 61 were processed culinary ingredients and 986 were minimally processed foods."
		(b) Give reasons for non-participation at each stage	12	"Alcoholic drinks were excluded from this analysis as they are subject to other specific regulations (n = 35)."
		(c) Consider use of a flow diagram		
Descriptive data	14*	(a) Give characteristics of study participants (eg demographic, clinical, social) and information on exposures and potential confounders	N/A	
		(b) Indicate number of participants with missing data for each variable of interest	12	"Out of 3,105 total items in the database, 109 items were designated as outside the Nova classification (e.g., fish oil supplements and multivitamins), and 16 items had no corresponding item in the NDNS nutrient databank as previously reported.[42] Alcoholic drinks were excluded from this analysis as they are subject to other specific regulations (n = 35). [40]"
		(c) *Cohort study*—Summarise follow-up time (eg, average and total amount)	N/A	
Outcome data	15*	*Cohort study*—Report numbers of outcome events or summary measures over time	N/A	
		Case-control study—Report numbers in each exposure category, or summary measures of exposure	N/A	
		Cross-sectional study—Report numbers of outcome events or summary measures	N/A	
Main results	16	(*a*) Give unadjusted estimates and, if applicable, confounder-adjusted estimates and their precision (eg, 95% confidence interval). Make clear which confounders were adjusted for and why they were included	19-24	"Table 4 and Figure 1 report the proportion of PP and UPP identified as unhealthy according to each NPM…."
		(*b*) Report category boundaries when continuous variables were categorized	N/A	
		(*c*) If relevant, consider translating estimates of relative risk into absolute risk for a meaningful time period	N/A	

Continued on next page

Other analyses	17	Report other analyses done—eg analyses of subgroups and interactions, and sensitivity analyses	20	"Table 4: Percentage of processed and ultra-processed products identified as unhealthy‡ in the UK according to each nutrient profiling model, with 95% confidence intervals."
**Discussion**				
Key results	18	Summarise key results with reference to study objectives	26	"This is the first study to compare the performance and consistency of existing UK food systems for classification of unhealthy food, and three novel NPMs based on UK dietary guidance…"
Limitations	19	Discuss limitations of the study, taking into account sources of potential bias or imprecision. Discuss both direction and magnitude of any potential bias	29	"Limitations include the use of the Nova classification. Whilst the Nova classification is widely used, it is not without limitations and criticism.[39,40] …"
Interpretation	20	Give a cautious overall interpretation of results considering objectives, limitations, multiplicity of analyses, results from similar studies, and other relevant evidence	26-27	"Traffic Lights appear to have not improved the healthiness of retail food purchases in the UK.[64]…"
Generalisability	21	Discuss the generalisability (external validity) of the study results	29	"Strengths of this study include the nationally representative food and drink supply for the UK with the most up-to-date food composition data, and novel NPMs constructed based on well-evidenced UK dietary guidance that were comparable to FSA-NPM and

				Traffic Lights in terms of nutrients of consideration…”
**Other information**				
Funding	22	Give the source of funding and the role of the funders for the present study and, if applicable, for the original study on which the present article is based	31	“SD is supported by the National Institute for Health and Care Research UCLH Biomedical Research Centre (NIHR UCLH BRC), Rosetrees Trust and Medical Research Council (MR/N013867/1)…”

*Give information separately for cases and controls in case-control studies and, if applicable, for exposed and unexposed groups in cohort and cross-sectional studies.

Note: An Explanation and Elaboration article discusses each checklist item and gives methodological background and published examples of transparent reporting. The STROBE checklist is best used in conjunction with this article (freely available on the Web sites of PLoS Medicine at http://www.plosmedicine.org/, Annals of Internal Medicine at http://www.annals.org/, and Epidemiology at http://www.epidem.com/). Information on the STROBE Initiative is available at www.strobe-statement.org.
